# Supplementary material for: What challenges did junior doctors face while working during the COVID-19 pandemic? A qualitative study
Source: BMJ Open. 2021 Dec 13;11(12):e056122. doi: 10.1136/bmjopen-2021-056122 (PMC8671849; doi:10.1136/bmjopen-2021-056122)
Supplement: Author's manuscript [file bmjopen-2021-056122.draft_revisions.pdf]

BMJ Open is committed to open peer review. As part of this commitment we make the peer review history of every article we publish publicly available.

When an article is published we post the peer reviewers' comments and the authors' responses online. We also post the versions of the paper that were used during peer review. These are the versions that the peer review comments apply to.

The versions of the paper that follow are the versions that were submitted during the peer review process. They are not the versions of record or the final published versions. They should not be cited or distributed as the published version of this manuscript.

BMJ Open is an open access journal and the full, final, typeset and author-corrected version of record of the manuscript is available on our site with no access controls, subscription charges or pay-per-view fees (http://bmjopen.bmj.com).

If you have any questions on BMJ Open's open peer review process please email info.bmjopen@bmj.com

BMJ Open

What challenges did junior doctors face whilst working during the COVID-19 pandemic? A qualitative study

Journal:	*BMJ Open*
Manuscript ID	bmjopen-2021-056122
Article Type:	Original research
Date Submitted by the Author:	12-Aug-2021
Complete List of Authors:	Spiers, Johanna; University of Birmingham, College of Medical and Dental Sciences<br>Buszewicz, Marta; University College London, Research Department of Primary Care and Population Health<br>Chew-Graham, Carolyn; University of Keele<br>Dunning, Alice; Bradford Teaching Hospitals NHS Foundation Trust<br>Taylor, Anna; University of Leeds<br>Gopfert, Anya; Torbay Council<br>Van Hove, Maria; University of Exeter<br>Teoh, Kevin; Birkbeck University of London, Department of Organizational Psychology<br>Appleby, Louis; University of Manchester, Psychiatry & Behavioral Sciences<br>Martin, James; University of Birmingham, Institute of Applied Health Research<br>Riley, Ruth; University of Birmingham Institute of Applied Health Research; University of Birmingham, of Applied Health Research
Keywords:	COVID-19, MENTAL HEALTH, Depression & mood disorders < PSYCHIATRY, QUALITATIVE RESEARCH

SCHOLARONE™
Manuscripts

I, the Submitting Author has the right to grant and does grant on behalf of all authors of the Work (as defined in the below author licence), an exclusive licence and/or a non-exclusive licence for contributions from authors who are: i) UK Crown employees; ii) where BMJ has agreed a CC-BY licence shall apply, and/or iii) in accordance with the terms applicable for US Federal Government officers or employees acting as part of their official duties; on a worldwide, perpetual, irrevocable, royalty-free basis to BMJ Publishing Group Ltd ("BMJ") its licensees and where the relevant Journal is co-owned by BMJ to the co-owners of the Journal, to publish the Work in this journal and any other BMJ products and to exploit all rights, as set out in our licence.

The Submitting Author accepts and understands that any supply made under these terms is made by BMJ to the Submitting Author unless you are acting as an employee on behalf of your employer or a postgraduate student of an affiliated institution which is paying any applicable article publishing charge ("APC") for Open Access articles. Where the Submitting Author wishes to make the Work available on an Open Access basis (and intends to pay the relevant APC), the terms of reuse of such Open Access shall be governed by a Creative Commons licence – details of these licences and which Creative Commons licence will apply to this Work are set out in our licence referred to above.

Other than as permitted in any relevant BMJ Author's Self Archiving Policies, I confirm this Work has not been accepted for publication elsewhere, is not being considered for publication elsewhere and does not duplicate material already published. I confirm all authors consent to publication of this Work and authorise the granting of this licence.

For peer review only

What challenges did junior doctors face whilst working during the COVID-19 pandemic? A qualitative study

Johanna Spiers (corresponding author)
Institute of Applied Health Research
University of Birmingham
Birmingham
United Kingdom
johanna.spiers@gmail.com
Tel: 07966 763 364

Marta Buszewicz
Research Department of Primary Care and Population Health
University College London Medical School
London
United Kingdom

Carolyn A. Chew-Graham
School of Medicine
Keele University
Keele
United Kingdom

Alice Dunning
Yorkshire Quality and Safety Research Group
Bradford Institute for Health research
Bradford Teaching Hospitals NHS Foundation Trust
Bradford
United Kingdom

For peer review only

Anna K. Taylor

Leeds Institute of Health Sciences

Faculty of Medicine and Health

University of Leeds

Leeds

United Kingdom

Anya Gopfert

Torbay Council,

Torquay,

TQ1 3DR

Maria van Hove

The University of Exeter,

Exeter,

EX4 4PY

Kevin Rui-Han Teoh

Department of Organizational Psychology

Birkbeck, University of London

London

United Kingdom

Louis Appleby

Division of Psychology and Mental Health

School of Medicine

University of Manchester

Manchester

United Kingdom

James Martin

For peer review only

For peer review only - http://bmjopen.bmj.com/site/about/guidelines.xhtml

Institute of Applied Health Research
University of Birmingham
Birmingham
United Kingdom

Ruth Riley
Institute of Applied Health Research
University of Birmingham
Birmingham
United Kingdom

Keywords: Junior doctors, COVID-19, Occupational Stress, Mental Health, Working Conditions, Qualitative Methods

Word count: 3760

For peer review only - http://bmjopen.bmj.com/site/about/guidelines.xhtml

Consolidated criteria for reporting qualitative studies [COREQ]:

Developed from: Tong, A., Sainsbury, P., & Craig, J. [2007]. Consolidated criteria for reporting qualitative research [COREQ]: a 32-item checklist for interviews and focus groups. *International journal for quality in health care*, *19*[6], 349-357.

**No. Item**	**Guide questions/description**	**Reported on page #**
**Domain 1: Research team and reflexivity**		
Personal characteristics		
1. Interviewer/ facilitator	Which author/s conducted the interview or focus group?	Page 11
2. Credentials	What were the researcher's credentials? E.g. PhD, MD.	Page 11
3. Occupation	What was their occupation at the time of the study?	Page 11
4. Gender	Was the researcher male or female?	Page 11
5. Experience and training	What experience or training did the researcher have?	Page 11
Relationship with participants		
6. Relationship established	Was a relationship established prior to study commencement?	Page 11
7. Participant knowledge of interviewer	What did the participant know about the researcher? E.g. personal goals, reasons for doing the research.	Page 11
8. Interviewer characteristics	What characteristics were reported about the interviewer/facilitator? E.g. bias, assumptions, reasons and interests in the research topic	Page 12
**Domain 2: study design**		
Theoretical framework		
9. Methodological orientation and theory	What methodological orientation was stated to underpin the study? E.g. grounded theory, discourse analysis, ethnography, phenomenology, content analysis	Page 12

For peer review only - http://bmjopen.bmj.com/site/about/guidelines.xhtml

Participant sampling		
10. Sampling	How were participants selected? E.g. purposive, convenience, consecutive, snowball	Page 10
11. Method of approach	How were participants approached? E.g. face-to-face, telephone, mail, email	Page 10
12. Sample size	How many participants were in the study?	Page 11
13. Non-participation	How many people refused to participate or dropped out? Reasons?	None
Setting		
14. Setting of data collection	Where was the data collected? E.g. home, clinic, workplace	Page 11
15. Presence of non-participants	Was anyone else present besides the participants and the researchers?	No
16. Description of the sample	What were the important characteristics of the sample? E.g. demographic data, date	Page 11
Data collection		
17. Interview guide	Were questions, prompts, guides provided by the authors? Was it pilot tested?	Page 11
18. Repeat interviews	Were repeat interviews carried out? If yes, how many?	No
19. Audio/visual recording	Did the research use audio or visual recording to collect the data?	Page 11
20. Field notes	Were field notes made during and/or after the interview or focus group?	Page 11
21. Duration	What was the duration of the interviews or focus group?	Page 11
22. Data saturation	Was data saturation discussed?	Page 12
23. Transcripts returned	Were transcripts return to participants for comment and/or correction?	No, due to lack of resources
**Domain 3: analysis and findings**		
Data analysis		
24. Number of data coders	How many data coders coded the data?	Page 12
25. Description of the coding tree	Did authors provide a description of the coding tree?	No
26. Derivation of themes	Derived from the data?	Page 12

For peer review only - http://bmjopen.bmj.com/site/about/guidelines.xhtml

27. Software	What software, if applicable, was used to manage the data?	Page 12
28. Participant checking	Did participants provide feedback on the findings?	No, due to lack of resources
Reporting		
29. Quotations presented	Were participant quotations presented to illustrate the themes/findings? Was each quotation identified? E.g. participant number	Pages 13-22
30. Data and findings consistent	Was there consistency between the data presented and the findings	Yes, see sages 13-22
31. Clarity of major themes	Were major themes presented in the findings?	Yes
32. Clarity of minor themes	Is there a description of diverse cases or discussion of minor themes?	Yes

For peer review only

For peer review only - http://bmjopen.bmj.com/site/about/guidelines.xhtml

What challenges did junior doctors face whilst working during the COVID-19 pandemic? A qualitative study

Abstract

Objectives: This paper reports findings exploring junior doctors' experiences of working during the COVID-19 pandemic in the UK.

Design: Qualitative study using in-depth interviews with 15 junior doctors. Interviews were audio-recorded, transcribed, anonymised and imported into NVivo 12 to facilitate data management. Data were analysed using reflexive thematic analysis.

Setting: NHS England.

Participants: A purposive sample of 12 female and three male junior doctors who indicated severe depression and/or anxiety on the DASS-21 questionnaire or high suicidality on Paykel's measure were recruited. These doctors self-identified as having lived experience of distress due to their working conditions.

Results: We report three major themes. Firstly, the challenges of working during the COVID-19 pandemic, which were both personal and organisational. Personal challenges were characterised by helplessness and included the trauma of seeing many patients dying, fears about safety and being powerless to switch off. Work-related challenges revolved around change and uncertainty, and included increasing workloads, decreasing staff numbers and negative impacts on relationships with colleagues and patients. The second theme was strategies for coping with the impact of COVID-19 on work, which were also both personal and organisational. Personal coping strategies were problem and emotion-focused, while several participants appeared to have moved from coping towards learned helplessness. Some organisations reacted to COVID-19 collaboratively and flexibly. Thirdly, participants reported a positive impact of the COVID-19 pandemic on working practices, which included

For peer review only - http://bmjopen.bmj.com/site/about/guidelines.xhtml

simplified new ways of working – such as consistent teams and longer rotations – as well as increased camaraderie and support.

Conclusions: Junior doctors described a variety of challenges whilst working during the pandemic. Coping strategies developed were both personal and organisational, and some changes in work were positive. We recommend that, post-pandemic, junior doctors are assigned to consistent teams and offered ongoing support.

Article summary: Strengths and limitations

- Participants were interviewed at the peak of the second wave of COVID-19 during the UK, meaning transcripts contain data that are highly relevant to the research question
- In-depth, reflexive thematic analysis was carried out on the data, leading to the development of rich, insightful themes
- Female participants outnumbered male participants in this study, potentially leading to gender imbalance
- Additionally, the wider study was not initially designed to explore experiences of working during COVID-19. Instead, participants naturally discussed this topic during interviews.

Funding statement

The study was funded by NIHR Research for Patient Benefit (grant number PB-PG-0418-20023). Please note that CCG is part-funded by West Midlands ARC.

Competing interests

None

For peer review only - http://bmjopen.bmj.com/site/about/guidelines.xhtml

Introduction

Doctors are more vulnerable to mental illnesses [such as anxiety and depression] and suicide than the general population [1, 2] In recent years, including those before the COVID-19 pandemic, UK doctors have reported understaffing, stretched resources, increased workload and burnout [3-7].

There is an additional need to attend to frontline workers' wellbeing during health crises [6-8]. Frontline workers caring for COVID-19 patients have reported stress and distress due to the strain on healthcare systems [9]. Such stressors include the need for rapid training around treating a new illness [9] and the psychological impact of exposure to unprecedented levels of suffering and COVID-19-related deaths, both of patients and colleagues [8, 10, 11].

These stressors led to healthcare professionals (HCP) reporting fears about contracting or spreading the virus as well as uncertainty due to new ways of working [11, 12]. Impacts of these fears and stressors include reduced sleep, self-harm, panic attacks, guilt, relationship breakdowns [11], concerns about lack of training [7] and psychological trauma [10].

The UK reported some of the highest numbers of COVID-19 cases in Europe [7]. In a recent paper, almost half of the 224 UK doctors surveyed (from junior doctors to consultants) felt that their mental health had been harmed by the pandemic, while a third reported impacts to their physical health [5]. Increased healthcare worker burnout is, therefore, a major concern at this time. We need a holistic understanding of the experiences and needs of frontline workers to mitigate psychological distress and burnout [11].

'Junior doctor' is the term given to qualified doctors who are still in training whilst working. They may have eight or more years of experience, depending on their speciality [13]. Junior doctors have reported fears that they will 'fail' or appear 'weak' if they take time off sick [14], making it harder for them to report mental health concerns [14]. This group faced unique challenges during COVID-19 due to uncertainties about exams [6], potential redeployment [8, 15, 16] and concerns about their learning opportunities [15, 16]. UK junior

For peer review only - http://bmjopen.bmj.com/site/about/guidelines.xhtml

doctors have reported that they did not receive enough education before treating COVID-19 patients [15]. They were also often faced with the difficult task of contacting patients' families to provide updates, since relatives were typically not permitted to visit [15].

Despite this, few researchers have looked in-depth at the psychological experiences of junior doctors. Instead, they have explored practical matters relating to this group, such as the resilience of new rotas (that is, assigning enough staff to cope with the workload) [17], redeployment [15, 16], the impact on training [18] and the provision of certain services such as obs and gynae [19].

Researchers have posited the need for more in-depth qualitative analysis in this area [5, 11]. This paper is part of a wider study [20, 21] designed to explore the impact of working conditions and cultures on junior doctors in general. Data collection coincided with the second wave of the pandemic in the UK, meaning the topic naturally arose for all 15 participants interviewed. As such, we aim to address this crucial gap in the literature and reflect the experiences of junior doctors working within the context of COVID-19.

Method

Study design and setting

This qualitative study is part of a larger mixed-methods study exploring junior doctors' perceptions of stress and distress. Semi-structured interviews were used to explore junior doctors' experiences of working during COVID-19. The study setting was the NHS in England.

Sampling and recruitment

A total of 456 junior doctors were initially recruited for an online survey exploring working cultures, psychological distress and suicidality between November 2020 and March 2021. They self-identified as participants, accessing the survey through posts on social media, junior doctor forums and via emails sent from their speciality schools. Survey participants whose results indicated severe depression and/or anxiety on the DASS-21 questionnaire [22] or high suicidality on Paykel's measure [23] were purposively contacted via email to ask if they would like to take part in an in-depth, qualitative survey. Interested individuals

For peer review only - http://bmjopen.bmj.com/site/about/guidelines.xhtml

contacted JS and gave informed consent. Participants were given the chance to ask JS questions about the research team and the study before interviews went ahead. Fifteen junior doctors (12 female, three male) were recruited.

Data collection

A semi-structured interview guide was developed by the research team and modified iteratively as data collection and analysis progressed. This guide aimed to capture participants' views, experiences, feelings and beliefs about working conditions and cultures which were perceived as stressful or distressing. The guide was informed by the existing literature, input from junior doctors on the study team as well as patient and public involvement (PPI) consultation exercises conducted before obtaining funding. Following conventions for semi-structured interviews [24], points from the topic guide were followed up with individualised questions exploring topics of interest and importance for each participant.

Interviews were conducted either on the telephone or via video call, from participants' homes or places of work. They took place between December 2020 and February 2021 – that is, during the second wave of the COVID-19 pandemic in the UK – and at a date and time that were convenient to the participants. A risk protocol was used to ensure appropriate support was provided to participants in the event of the disclosure of suicidal ideation. The in-depth interviews were conducted by JS, a female PhD psychologist with extensive qualitative methods expertise. JS also recorded any pertinent observations in field notes following each interview. Interviews ranged from 29 minutes to 102 minutes in length (mean = 62.8 minutes).

The audio-recorded interviews were transcribed verbatim and checked for accuracy by JS before analysis. All transcripts were anonymised before discussion within the wider research team. Reflexive notes were recorded by researchers throughout the process.

For peer review only - http://bmjopen.bmj.com/site/about/guidelines.xhtml

Patient and public involvement and engagement

There are three junior doctors on the research team, all of whom consulted with other colleagues in the PPI team about the initial research idea and participated in analysis meetings. Five junior doctors gave feedback on the initial funding application, while four fed back on the protocol, topic guide and participant-facing documents.

Data analysis

Data were analysed by JS using reflexive thematic analysis [25, 26]. An inductive, explicit, critical realist approach was adopted since this was in line with the researchers' desire for a rich, data-driven analysis. Data saturation is not a relevant concept within this type of approach, in which it is accepted that each new participant adds fresh insights. Analysis began once all interviews had been conducted. Transcripts were analysed one by one using NVivo 12. As analysis progressed, a table of themes was generated and refined. Each new transcript led to new codes and themes being added or expanded. In addition, four members of the team (RR, MB, AT, CCG) read and fed back on six of the 15 interviews. Their views and insights were collaboratively incorporated into the NVivo codes. JS then refined these codes to create relevant tables of themes once all interviews had been analysed and discussed. Analysis continued and deepened during the write-up, where shared meanings were generated and described for each theme [26].

Reflexivity

RR, the study PI, is epistemological steeped in qualitative traditions underpinned by interpretivism, and phenomenology, and is oriented by critical theory such as feminism. With a background in psychology and sociology, and as a non-clinician, RR's interest in work cultures and conditions may also have been influenced by her experience of working as a researcher and medical educator, where she has observed rationalist and hegemonic cultures with an intolerance of vulnerability. Such orientations are likely to have influenced this topic and an interest in exploring why female doctors are more likely to experience distress.

For peer review only - http://bmjopen.bmj.com/site/about/guidelines.xhtml

JS, the lead analyst on this paper, is a qualitative health psychologist. She has an interest in in-depth, interpretative methods and so may have been influenced to see nuanced psychological interpretations of data. In addition, she is white, cis-gendered, heterosexual and able-bodied. This heteronormative positioning is likely to have impacted her interviewing and analysis.

Findings

All fifteen participants discussed the impact of COVID-19 on their working conditions. Findings divided into three major themes: Challenges of working during the COVID-19 pandemic; Strategies for coping with the impact of COVID-19 on work; Positive impact of the COVID-19 pandemic on working practices.

See Table One for an overview of all relevant themes and subthemes.

For peer review only

Theme	Subtheme
Challenges of working during the Covid-19 pandemic<br>*"patients were just dying in front of us so quickly and they were young" (P5)*	Personal impact
	Work-related impact
Strategies for coping with the impact of Covid-19 on work<br>*"So although I should have moved on from GP I ended up staying in GP so I was actually there for eight months." (P7)*	Personal strategies
	Organisational strategies
Positive impact of Covid-19 on working practices<br>*"since COVID, things have improved slightly there's, um we have something called like the rest and recuperation hub." (P6)*	Positive new ways of working
	Additional support and camaraderie

Table one: Table of themes for junior doctors' experience of working during the Covid-19 pandemic

For peer review only - http://bmjopen.bmj.com/site/about/guidelines.xhtml

Challenges of working during the COVID-19 pandemic

Participants described challenges related to their work as junior doctors during the COVID-19 pandemic. Challenges were personal or work-related.

Personal impact

Working as junior doctors during the COVID-19 pandemic affected participants' mental health. Throughout this theme, there is a sense that participants felt helpless and powerless as they strove to carry out their jobs in such unmapped territories.

For peer review only

P5 described the harmful impact of being exposed to death and suffering:

> *I'd seen [pause] a whole ward just emptied out and then refilled overnight, after people had just died. It was horrendous. Uh, I was like, "I need to talk to somebody about this or I'm just going to go home and cry". (P5, female)*

Participants felt helpless in the face of fears for their own safety and that of their loved ones. Initially, they were unsure of how to protect themselves or of the risk they might pose to their families:

> *…we had someone that we thought was, um, COVID, but it was very, very early on. And I remember being told off for wearing a mask. (P3, female)*

> *…we were worried about if we were taking home our clothes, if we were making other people sick, if we would get sick, it was an incredibly stressful working environment. (P5, female)*

As time went on, fears for personal safety came from different sources, with P10 reporting that her colleagues were not maintaining safety standards. However, as a junior doctor, she felt powerless to ask for this to change:

> *It's not patients, it's staff. I find that really stressful. Like you walk past an office and there might be two or three people sat in an office having a chat, all with their masks*

under their chin. […] I don't feel confident enough to knock on the window and be like guys, what are you doing? But I know that them doing it puts me more at risk and puts the patients at risk. […] You see stuff being wrong and you're like every day, like multiple times a day you're like do I say something, do I not say something? And you feel bad for not saying something. (P10, female)

P10's description of this discomfort could be defined as moral injury; that is, the distress that occurs when a person witnesses or carries out an act that is contrary to their values.

Participants were powerless to switch off or rest when they got home from work:

You couldn't switch off, you never felt like you'd had, uh, done a good job. (P5, female)

…my sleep is awful again, I'm waking up, I think COVID hasn't helped with these sort of flashbacks. (P1, female)

Participants did not feel clinically supported in the new working environment caused by COVID-19, which led to further powerlessness and stress. The lack of support was both educational:

I'm going to personally take responsibility for changing […] the big scary machine that I'm not trained in, and, uh, figure out how it works, whilst the patient is there trying to physically die in front of me, but so are five others, so oh well, no help is coming. (P5, female)

And psychological:

…they got some psychologists who would be available and very occasionally they would come on the ward. [pause] And they would talk to the nurses. And that was it. No. It felt assumed to be on the nurses and people working in ITU and just ordinary junior doctors didn't [pause] didn't seem to matter. (P14, female)

For peer review only - http://bmjopen.bmj.com/site/about/guidelines.xhtml

Perceived poor communication meant that participants felt unsupported in various ways. One felt her safety was compromised:

> *…you will turn up on a ward and you will find out halfway through handover that they've had a positive case over the weekend. (P10, female)*

Additionally, a junior doctor whose family were overseas reported feeling unsupported by her hospital after contracting COVID-19:

> *…when I went to quarantine, I realised that no-one actually cares about you from the hospital? […] No-one called me! […] When I was very very sick, imagine that, if I had, if I had literally no-one. (P4, female)*

Work-related impact

The work-related impact of working during a COVID-19 context centred around uncertainty and change. These included changes to workload, staffing levels, relationships with colleagues and patients, lack of support and uncertainty around new ways of working.

Workload changed by growing when COVID-19 hit, leading to further stressors.

> *…on a Friday in the middle of the day when there was no consultant around […] I gained 14 new patients who I'd not met before […] that was a really stressful day. (P6, female)*

Workload increased out of hours as well, as participants had to learn about the virus:

> *So we were getting 20 emails a day, and every single one would have a red flag saying "vital, important, must read", and you'd worry you'd missed something […] there's so much information, it was constant, and you couldn't switch off, because it would impact your job. (P5, female)*

For peer review only

As workload rose, staffing levels, which had already been stretched, changed by reducing further due to staff illness or the need to self-isolate:

So it's very very short-staffed because a lot of the people are self-isolating, ill with COVID, or just because you know they've worked already five or six days in a row, and obviously they're quite tired and they have to take a break. (P12, male)

The additional workload changed working relationships in various ways. Participants reported that colleagues became irritable or verbally aggressive due to increased stress:

I think everyone got a little bit more [pause], um, maybe snippy? With each other? 'Cause we were all are very stressed and anxious. (P3, female)

…a registrar wearing an MF53 mask[1] *and the consultant laying into him basically shouting at him that […] he was depriving someone else who actually needed this mask […] emotions were running high because people were scared. (P14, female)*

One trainee, based in general practice, reported that patients had become abusive during telephone appointments:

…sometimes people lose sense of the fact that it's another human being on the end of the phone with them. (P7, female)

That participant also reported finding the change to telephone appointments clinically challenging:

I don't think you realise how much you rely on seeing someone in front of you to know how well they are. And talking to someone over the phone it just feels a lot more dangerous. (P7, female)

[1] This is a full face, military style of mask

For peer review only - http://bmjopen.bmj.com/site/about/guidelines.xhtml

Compounding these conflicts was the fact that it became harder to speak with and get support from peers due to the safety measures:

> *Um, and now with COVID where you're only allowed, like, two people in each room, it, it's very difficult to, um, socialise and talk. (P8, female)*

Participants also found the uncertainty around changes to rotas and exams challenging:

> *…a fair amount of uncertainty and the problem this time is that, ah, a lot of courses are still going ahead, exams are still going ahead, but we've been moved onto emergency rotas with a week's notice. (P8, female)*

Anxiety due to uncertainty about redeployment was reported:

> *…quite anxious and uncertain about whether that was going to happen and would sort of check my emails pretty consistently to see whether that was actually whether that was going to be um delayed or stopped because of COVID redeployment. (P6, female)*

The pandemic meant that new ways of working were quickly developed and implemented. Trying to adjust to these changes was another challenge. One trainee in psychiatry talked about the challenges of working from home:

> *…you've not got those people around you to bounce things off, so you might get an email and it might be quite an anxiety-inducing email because it might say someone's suicidal, you need to see them, and you're thinking, oh, I can't see them, erm, and normally, kind of in an office you'd just be able to ask, can anyone else see them? (P2, female)*

Strategies for coping with the impact of COVID-19 on work

For peer review only - http://bmjopen.bmj.com/site/about/guidelines.xhtml

Participants described both personal and organisational strategies for coping with the above challenges.

Personal coping strategies

Problem-focused and emotion-focused coping strategies were utilised for dealing with the challenges of COVID-19. Problem-based, or practical, coping strategies included seeking professional therapy:

> *…when lockdown came back in […] I noticed that like I was feeling low so I referred myself to the Let's Talk Wellbeing, erm which is like the community, CBT, GP, self-referral system. And I found that really helpful erm so that kind of stopped me spiralling. (P10, female)*

Another participant volunteered to take on the work of calling relatives to let them know their loved ones were very sick, perhaps to regain some control:

> *I used to volunteer to kind of be the person making those phone calls, cos it was, it felt like you were able to do something about it at least? It wasn't that sort of like, "I put lines in people and hopefully", and then just watching them die. (P5, female)*

Emotion-based coping strategies included crying:

> *So I cried a lot outside. Because it was getting warmer so you could go outside. Hug a tree, cry. (P14, female)*

And stoicism, although this latter strategy suggested a sense of resignation:

> *And [pause] and in a way it didn't really matter that our rota changed, because there was nothing else to do? (P8, female)*

For peer review only - http://bmjopen.bmj.com/site/about/guidelines.xhtml

Or perhaps even learned helplessness in the face of such trauma:

> *I think erm you know everyone's a bit more sort of resigned to things now and it feels like we've sort of erm entered a collective sort of depressive state of acceptance. (P9, male)*

Organisational strategies

Just as participants found ways to cope with the challenges of COVID-19, so did the organisations and teams for whom they worked, with some trusts and teams demonstrating collaborative, flexible thinking. One participant reported flexibility for colleagues who had to self-isolate:

> *...most of the places have let the person sort of choose whether they you know, if it's your child that's got a fever and actually you know you're isolating and could do things then that's fine. But if you're poorly then you're poorly and that's fine. (P11, female)*

P14 described the need for her team to make pragmatic decisions about how to treat COVID-19 patients:

> *So if someone was clearly dying, they would [pause] be stepped down to a normal ward because on a normal ward they could at least have a visitor for one hour a day. (P14, female)*

P5 reported that her team pulled together to help one another in the new circumstances:

> *...there'd be so many [emails] even coming in during our shift, we'd divide it up, so we'd say, "you read these five, I'll read these five, you read these five, and then I'll read these five", and then we'd kind of share what we've learned from them. (P5, female)*

For peer review only - http://bmjopen.bmj.com/site/about/guidelines.xhtml

Positive impact of COVID-19 on working practices

Participants reported that working as junior doctors during the COVID-19 pandemic had some positive impacts. These were new ways of working as well as additional support and camaraderie.

Positive new ways of working

These positive changes appeared to revolve around a simplified way of working, which included consistent teams, longer rotations and less red tape.

Several participants reported that they were now working in a consistent team, rather than regularly working with new colleagues. This was experienced as positive:

> *So normally, you're kind of working with somebody new every day almost. But we worked in teams that didn't rotate, so you had […] this team that you worked with very intensely for those four months as well, and that support structure was really good. (P5, female)*

> *…we got really to know each other, we had a little social WhatsApp group where we'd, like, post pictures of the cakes we were gonna bring in, you know, everyone bought in food. We almost looked forward to going to work because you were like, oh, my buddies are there. (P8, female)*

Rotations were paused for many junior doctors. Although this could lead to uncertainty, as reported in the previous theme, it also had some positive impacts:

> *So we were on the first rotation for four months and then the second for eight months […] Um, so, I guess it would have depended on what ward you got stuck on? [interviewer laughs] Um, I got stuck on one of the nice placements, I really enjoyed myself on the ward. (P3, female)*

For peer review only - http://bmjopen.bmj.com/site/about/guidelines.xhtml

Various practical changes to working patterns were also experienced as positive. These included the ability to work from home and reduced red tape:

> *…just get away and do something relaxing, even if it's just go for a walk around the local canal and come back on a lunchtime is so much more achievable when you're working from home. So I think that's been really good. (P2, female)*

> *…they say oh, we want you to travel to a hospital on your day off to show us your passport and your GMC certificate. And it's like I've been – doing this for 10 years. I've worked for you six times! Like, you've got my details [both laugh]. And that's one thing where COVID has been really good because now they do it online and I'm like, why couldn't you have always done this? (P8, female)*

One participant even appeared to cite COVID-19 as a motivator for returning to work at the NHS after time in another career:

> *…then COVID came and I wanted to come back to medicine anyway so I thought okay fine then let's just crack on with erm with the NHS. (P15, female)*

Additional support and camaraderie

Some participants reported that new supportive measures had been put in place by their workplaces:

> *And since COVID, things have improved slightly, there's, um we have something called like the rest and recuperation hub, which is like a room erm that does free teas and coffees and a few snacks […] you go there on your breaks to relax. (P6, female)*

P1 reported that her hospital made an effort to offer junior doctors support, although this was against the backdrop of a toxic working environment:

For peer review only - http://bmjopen.bmj.com/site/about/guidelines.xhtml

I'd say the culture's getting worse except for the fact that they send an e-mail out every now and again with some contact numbers and that's what COVID has done. (P1, female)

It should be noted that the reports of improved support were tempered – note that participants reported 'slight' improvements to a culture that was also 'getting worse'.

One participant stated that her hospital offered practical support in the form of food during the first wave of the virus:

…they provided hot meals, which, at the beginning, when there were huge queues at the supermarket, and we were working 12-hour shifts, five days a week, and, um, [pause] and it was unpredictable whether you could kind of get food, because there were a lot of shortages and things. (P5, female)

Discussion

Fifteen junior doctors were interviewed between December 2020 and February 2021 about their perceptions of stress and distress in their workplace cultures. All participants discussed how COVID-19 impacted their experiences. Three major themes were generated: Challenges of working during the COVID-19 pandemic; Strategies for coping with the impact of COVID-19 on work; Positive impact of the COVID-19 pandemic on working practices. These will now be discussed in the light of existing literature.

Participants reported that working during COVID-19 resulted in feelings of sadness, moral injury, being out of control and unsupported. Similarly, previous researchers have reported fear, anxiety, depression, exhaustion and burnout amongst frontline workers at this time [8, 9, 11, 16].

One participant described how hard it was to see so many patients dying. Others [8, 10, 27] have cited grief and managing death as especially challenging. We suggest that newer junior

For peer review only - http://bmjopen.bmj.com/site/about/guidelines.xhtml

doctors might need extra support to process grief given their relative lack of exposure to death.

Participants also described existential fears about their safety and that of their loved ones. Such fears have frequently been reported during this time [6, 10, 15], with some HCP living away from home to protect their families during COVID-19 [11].

One participant reported an experience of moral injury in the context of her safety concerns regarding staff behaviour. Moral injury due to redeployment away from long-term patients [15] or concerns about letting patients down [8] during COVID-19 has been reported. Our findings add another layer, demonstrating that moral injury can also arise due to staff members neglecting safety protocols. Newman et al. [11] recommended support for moral injury during this time.

Participants felt unsupported whilst working in these new circumstances, a finding reflected elsewhere [7, 10, 11]. The need for extra training and support for junior doctors during the pandemic has been reported [15]. We echo this recommendation, suggesting that support can come from good leadership and a feeling of being valued within a team.

Participants reported that while their workload rose due to the pandemic, staffing levels decreased. Previous research has shown that UK HCP are already working in an under-resourced environment and that workload is a stressor [21, 28-30]. Crises such as COVID-19 highlight the need for extra resources for our healthcare system, echoing the recommendations of the 2009 Boorman report [31], which have been widely neglected [6]. Additionally, it is harder for frontline workers to take breaks during a pandemic [5, 10], adding to the potential for burnout since longer working hours are a risk factor [32]. These stressors also impacted relationships with both colleagues and patients [8].

Participants in the current study, like others [7, 10, 11], felt unsupported during this time. Some HCPs have refused or were reluctant to take on roles for which they did not feel qualified [8, 11, 16], whilst others did this work despite feeling unsupported [9, 16].

For peer review only - http://bmjopen.bmj.com/site/about/guidelines.xhtml

Cubitt and colleagues [5] have highlighted the need for rotas that enable well-being rather than merely being resilient. Qualitative research such as the current study adds depth to these recommendations by demonstrating the instability and overwork HCP face.

Participants used problem-focused coping strategies such as attending therapy or volunteering for certain roles to cope during COVID-19. Chinese nurses have reported volunteering for extra duties [9], demonstrating dedication despite the challenges. Emotion-focused strategies such as crying were reported in our study, whilst humour, rationalisation and distraction [9] as well as meditation and time with friends [11] have been reported elsewhere. Participants in the current study also reported stoicism which verged on learned helplessness, demonstrating that personal coping strategies alone are not enough. Junior doctors need organisational support especially, although not exclusively, during crises like COVID-19. However, the emphasis continues to be on individual rather than organisational interventions and coping strategies [33, 34].

Various individualised coping strategies have been suggested in the literature from the UK, including healthy eating, attending training, going to therapy, support networks [6] and 'wobble rooms' [15]. However, San Juan and colleagues [6] recognised that finding time for these activities might be difficult. Indeed, it could be posited that encouraging such strategies places the responsibility for managing an unmanageable system with individuals, rather than the systems themselves [6], despite COVID-19 having compounded existing stressors, severe workforce shortages and burnout [35]. It is argued that, in our neo-liberal culture, responsibility for wellbeing is often placed on the individual, exonerating the state and systems for the wellbeing of workers [36, 37]. Therefore, in line with San Juan et al. [6], we recommend a focus on organisational coping strategies.

Participants reported that their teams responded to COVID-19 flexibly, making pragmatic decisions about patient care and working as a team. San Juan and colleagues [6] recommended leadership, communication, peer support and flexibility as organisational tools which could be used to help during a pandemic. Improved communications were also emphasised by Coughlan et al. [15]. Given the observation that poor communications during COVID-19 increased stress levels, this appears to be an important area.

For peer review only - http://bmjopen.bmj.com/site/about/guidelines.xhtml

Participants reported several potentially positive impacts of working during the pandemic. These included working in more consistent teams. San Juan and colleagues [6] have similarly reported that consistent teams are helpful for HCP, while inconsistent teams make it harder for junior doctors to seek support [14], increasing stress and vulnerability to mental ill-health [21]. As such, we recommend that, where possible, policymakers consider the use of consistent teams for junior doctors going forward.

While both benefits and disadvantages of working from home were reported in our study, disadvantages have been reported elsewhere, especially for HCP with young children or work that includes confidential and sensitive meetings [8]. The reduction in red tape reported by one participant appears to be a novel finding. We would suggest that any such reductions should be maintained after the pandemic ends, as this will reduce time pressures for junior – and senior – doctors.

Participants stated that new supportive measures, such as 'wobble rooms' had been put into place during COVID-19. Such spaces have been deemed helpful by other researchers [7, 10, 15], although there are anecdotal reports that many of these spaces have now been closed as hospitalisations from COVID-19 reduce. In contrast, some HCP reported that, rather than being provided with 'wobble rooms', the extra strain on the system meant that there were fewer places than usual to shower, rest or relax with colleagues [5, 6].

In line with our findings, Vindrola-Padros et al. [7] reported that there was extra signposting towards support during COVID-19; however, there was not often time to engage with this support. Additionally, it has been anecdotally reported that much of this support has been withdrawn now. This adds further weight to the notion that systemic, holistic changes are needed to support NHS staff, rather than individual ones [6].

Limitations

This study has various strengths, including being the first qualitative paper (to our knowledge) to explore the experiences of junior doctors during COVID-19. Our data was

For peer review only - http://bmjopen.bmj.com/site/about/guidelines.xhtml

collected during the pandemic and we utilised in-depth, collaborative thematic analysis. However, despite these strengths, the paper has several limitations. We did not recruit these participants specifically to talk about the Covid-19 pandemic. Rather, the timing of the study meant that the topic arose naturally. As such, the interview guide could have been designed to ask participants more thoroughly about these experiences. Additionally, some of the junior doctors had more experience of working with COVID-19 patients than others, meaning some participants are better represented in this paper than others. Further, there is a notable gender disparity, with a higher proportion of female doctors taking part. More female (n=12) participants volunteered than males (n=3). The increased willingness of female participants to speak about their experiences may reflect evidence indicating that female doctors are more likely to experience distress and end their lives [1]. The higher proportion of female participants may also reflect gendered help-seeking behaviour for mental ill-health, evidenced in the wider population [38], as well as the fact that female doctors are more likely to take part in research than their male counterparts [39].

Conclusions and recommendations

We conclude that junior doctors working during the COVID-19 pandemic faced multiple stressors and used various coping mechanisms to deal with these, with greater or lesser degrees of success. Several unexpected benefits of this period arose, including new ways of working and additional support and camaraderie. We believe that the responsibility for alleviating the stress and distress of junior doctors working during times of stress lies with systems of employment and systemic workforce gaps, rather than with individuals. As such, we recommend holistic, system-wide changes such as better communication strategies, increased flexibility around home-based working and other logistical issues as well as stronger, more compassionate leadership going forward. Additionally, we suggest that, where possible, junior doctors are assigned to consistent teams and offered fuller psychological support.

Author contributions: RR, JS, CCG, MB, AD, KT, AG, AT, MvH, LA, JM – substantial contributions to conception and design; approval of final version to the published.

For peer review only - http://bmjopen.bmj.com/site/about/guidelines.xhtml

RR, JS, CCG, MB, AT, KT, AD, AT – acquisition, analysis or interpretation of data; drafting the article or revising it critically for important intellectual content.

Acknowledgements: The authors would like to thank all junior doctor participants who kindly gave their time to share their experiences of working in the NHS. We also wish to thank members of the PPIE group who provided valuable input throughout the study.

Data sharing: This study has not received ethical approval to share confidential data with any third party other than the research team.

Ethical approval: Ethical approval was granted by the University of Birmingham and Health Research Authority (reference number: 19/HRA/6579)

For peer review only

For peer review only - http://bmjopen.bmj.com/site/about/guidelines.xhtml

References

1. Dutheil, F., et al. *Suicide among physicians and health-care workers: A systematic review and meta-analysis*. PloS one. 2019. **14**(12): p. e0226361

2. Petrie, K., et al. *Interventions to reduce symptoms of common mental disorders and suicidal ideation in physicians: A systematic review and meta-analysis*. The Lancet Psychiatry. 2019. **6**(3): 225-34.

3. Brooks, S. K., et al. *Review of literature on the mental health of doctors: Are specialist services needed?* Journal of Mental Health. 2011. **20**(2): 146-56.

4. Chopra, S. S. et al. *Physician Burnout*. JAMA. 2004. **291**(5): 633–33.

5. Cubitt, L. J. et al. *Beyond PPE: A mixed qualitative-quantitative study capturing the wider issues affecting doctors' well-being during the COVID-19 pandemic*. BMJ Open. 2021. **11**(3): e050223.

6. San Juan, N. V., et al. *Healthcare Workers' mental health and wellbeing during the COVID-19 pandemic in the UK: Contrasting guidelines with experiences in practice*. MedRxiv. 2020; advanced online.

7. Vindrola-Padros C., et al. *Perceptions and experiences of healthcare workers during the COVID-19 pandemic in the UK*. BMJ Open. 2020; **10**(11): Advanced online: e040503.

8. Liberati E., et al. *A qualitative study of experiences of NHS mental healthcare workers during the Covid-19 pandemic*. BMC Psychiatry. 2021. **21**(1): 1-12.

9. Sun, N., et al. *A qualitative study on the psychological experience of caregivers of COVID-19 patients.* American Journal of Infection Control. 2020. **48**(6): 592-8.

10. Blake, H., et al. *COVID-well study: Qualitative evaluation of supported wellbeing centres and psychological first aid for healthcare workers during the COVID-19 pandemic*. International Journal of Environmental Research and Public Health. 2021. **18**(7): 3626-59.

11. Newman K. L., et al. *Experiences and emotional strain of NHS frontline workers during the peak of the COVID-19 pandemic*. International Journal of Social Psychiatry. 2021; advanced online 00207640211006153.

12. Cawcutt K. A., et al. *Fighting fear in healthcare workers during the COVID-19 pandemic*. Infection Control & Hospital Epidemiology. 2020. **41**(10): 1192-3.

13. BMA, *https://www.bma.org.uk/advice-and-support/international-doctors/life-and-work-in-the-uk/toolkit-for-doctors-new-to-the-uk/doctors-titles-explained.* 2020.

For peer review only

For peer review only - http://bmjopen.bmj.com/site/about/guidelines.xhtml

14. Fox F. E., et al. *Junior doctors' experiences of personal illness: A qualitative study*. Medical Education. 2011. **45**(12): 1251-61.

15. Coughlan C., et al. *COVID-19: Lessons for junior doctors redeployed to critical care*. Postgraduate Medical Journal. 2021. **97**(1145): 188-91.

16. Lion P., et al. *ENT trainees' experience of redeployment during the coronavirus disease 2019 pandemic: A qualitative study*. The Journal of Laryngology & Otology, 2021. 1-13.

17. Aziz, A., et al. *Rapid transformation of the medical on-call rotas to deal with the COVID-19 pandemic: A case study*. Future Healthcare Journal. 2021; **8**(1): e36.

18. Seifman M. A., et al. *COVID-19 impact on junior doctor education and training: A scoping review*. Postgraduate medical journal. 2021.

19. Rimmer, M. P., et al. *Provision of obstetrics and gynaecology services during the COVID-19 pandemic: A survey of junior doctors in the UK National Health Service*. BJOG: An International Journal of Obstetrics & Gynaecology. 2020. **127**(9): 1123-8.

20. Riley, R., et al. *Protective factors and sources of support in the workplace as experienced by UK foundation and junior doctors: A qualitative study*. BMJ Open. 2021. **11**(6): e045588.

21. Riley, R., et al. *Sources of work-related psychological distress experienced by UK-wide foundation and junior doctors: A qualitative study*. BMJ Open. 2021. **11**(6): e043521.

22. Osman, A., et al. *The depression anxiety stress scales—21 [DASS-21]: Further examination of dimensions, scale reliability, and correlates*. Journal of clinical psychology. 2012. **68**(12): 1322–1338.

23. Paykel, E. S., et al. *Suicidal feelings in the general population: A prevalence study.* The British Journal of Psychiatry. 1974. **214**(582): 460–9.

24. King N. and C. Horrocks, *Interviews in Qualitative Research*. 2010, London: Sage.

25. Braun V. and V. Clarke, *Using thematic analysis in psychology*. Qualitative research in psychology, 2006. **3**(2): 77-101.

26. Braun V. and V. Clarke, *Reflecting on reflexive thematic analysis*. Qualitative Research in Sport, Exercise and Health, 2019. **11**(4): 589-97.

27. Johnson, S., et al. *Impact on mental health care and on mental health service users of the COVID-19 pandemic: A mixed methods survey of UK mental health care staff*. Social Psychiatry and Psychiatric Epidemiology, 2021. **56**(1): 25-37.

For peer review only - http://bmjopen.bmj.com/site/about/guidelines.xhtml

28. Kinman G. and K. Teoh K, *What could make a difference to the mental health of UK doctors? A review of the research evidence*. 2018.

29. Kinman, G., et al. *The mental health and wellbeing of nurses and midwives in the United Kingdom*. Royal College of Nursing Foundation and the Society for Occupational Medicine, 2020.

30. Oliver, D., *The NHS's understaffing is its Achilles' heel*. BMJ: British Medical Journal (Online), 2017. 357.

31. Boorman, S. *NHS Health and well-being review: Interim report*. NHS Health and Wellbeing Review, 2009.

32. Amoafo, E., et al. *What are the significant factors associated with burnout in doctors?* Occupational Medicine, 2015; **65**(2): 117-21.

33. Pollock, A., et al. *Interventions to support the resilience and mental health of frontline health and social care professionals during and after a disease outbreak, epidemic or pandemic: A mixed methods systematic review*. Cochrane Database of Systematic Reviews, 2020. 11.

34. Sood, A., et al. *Stress management and resilience training among department of medicine faculty: A pilot randomized clinical trial*. Journal of general internal medicine, 2011. **26**(8): 858-61.

35. Royal College of Surgeons of Edinburgh, https://committees.parliament.uk/writtenevidence/11033/html/, 2020.

36. Joseph, J., *Resilience as embedded neoliberalism: A governmentality approach*. Resilience, 2013. **1**(1): 38-52.

37. Traynor, M. *Guest editorial: What's wrong with resilience*. Journal of Research in Nursing, 2018. **23**(1): 5-8.

38. Seidler, Z. E., et al. *The role of masculinity in men's help-seeking for depression: A systematic review*. Clinical Psychology Review, 2016. **49**: 106-18.

39. Cull, W. L., et al. *Response rates and response bias for 50 surveys of pediatricians.* Health Services Research, 2005. **40**(1): 213-26.

For peer review only

BMJ Open

What challenges did junior doctors face whilst working during the COVID-19 pandemic? A qualitative study

Journal:	*BMJ Open*
Manuscript ID	bmjopen-2021-056122.R1
Article Type:	Original research
Date Submitted by the Author:	06-Oct-2021
Complete List of Authors:	Spiers, Johanna; University of Birmingham, College of Medical and Dental Sciences<br>Buszewicz, Marta; University College London, Research Department of Primary Care and Population Health<br>Chew-Graham, Carolyn; University of Keele<br>Dunning, Alice; Bradford Teaching Hospitals NHS Foundation Trust<br>Taylor, Anna; University of Leeds<br>Gopfert, Anya; Torbay Council<br>Van Hove, Maria; University of Exeter<br>Teoh, Kevin; Birkbeck University of London, Department of Organizational Psychology<br>Appleby, Louis; University of Manchester, Psychiatry & Behavioral Sciences<br>Martin, James; University of Birmingham, Institute of Applied Health Research<br>Riley, Ruth; University of Birmingham Institute of Applied Health Research; University of Birmingham, of Applied Health Research
**Primary Subject Heading**:	Mental health
Secondary Subject Heading:	Health services research, Medical education and training, Qualitative research
Keywords:	COVID-19, MENTAL HEALTH, Depression & mood disorders < PSYCHIATRY, QUALITATIVE RESEARCH

SCHOLARONE™
Manuscripts

I, the Submitting Author has the right to grant and does grant on behalf of all authors of the Work (as defined in the below author licence), an exclusive licence and/or a non-exclusive licence for contributions from authors who are: i) UK Crown employees; ii) where BMJ has agreed a CC-BY licence shall apply, and/or iii) in accordance with the terms applicable for US Federal Government officers or employees acting as part of their official duties; on a worldwide, perpetual, irrevocable, royalty-free basis to BMJ Publishing Group Ltd ("BMJ") its licensees and where the relevant Journal is co-owned by BMJ to the co-owners of the Journal, to publish the Work in this journal and any other BMJ products and to exploit all rights, as set out in our licence.

The Submitting Author accepts and understands that any supply made under these terms is made by BMJ to the Submitting Author unless you are acting as an employee on behalf of your employer or a postgraduate student of an affiliated institution which is paying any applicable article publishing charge ("APC") for Open Access articles. Where the Submitting Author wishes to make the Work available on an Open Access basis (and intends to pay the relevant APC), the terms of reuse of such Open Access shall be governed by a Creative Commons licence – details of these licences and which Creative Commons licence will apply to this Work are set out in our licence referred to above.

Other than as permitted in any relevant BMJ Author's Self Archiving Policies, I confirm this Work has not been accepted for publication elsewhere, is not being considered for publication elsewhere and does not duplicate material already published. I confirm all authors consent to publication of this Work and authorise the granting of this licence.

For peer review only

What challenges did junior doctors face whilst working during the COVID-19 pandemic? A qualitative study

Johanna Spiers (corresponding author)

Institute of Applied Health Research

University of Birmingham

Birmingham

United Kingdom

j.spiers@bham.ac.uk

Tel: 07966 763 364

Marta Buszewicz

Research Department of Primary Care and Population Health

University College London Medical School

London

United Kingdom

Carolyn A. Chew-Graham

School of Medicine

Keele University

Keele

United Kingdom

Alice Dunning

Yorkshire Quality and Safety Research Group

Bradford Institute for Health research

Bradford Teaching Hospitals NHS Foundation Trust

Bradford

United Kingdom

For peer review only

Anna K. Taylor

Leeds Institute of Health Sciences

Faculty of Medicine and Health

University of Leeds

Leeds

United Kingdom

Anya Gopfert

Torbay Council,

Torquay,

TQ1 3DR

Maria van Hove

The University of Exeter,

Exeter,

EX4 4PY

Kevin Rui-Han Teoh

Department of Organizational Psychology

Birkbeck, University of London

London

United Kingdom

Louis Appleby

Division of Psychology and Mental Health

School of Medicine

University of Manchester

Manchester

United Kingdom

James Martin

For peer review only

Institute of Applied Health Research

University of Birmingham

Birmingham

United Kingdom

Ruth Riley

Institute of Applied Health Research

University of Birmingham

Birmingham

United Kingdom

Keywords: Junior doctors, COVID-19, Occupational Stress, Mental Health, Working Conditions, Qualitative Methods

For peer review only - http://bmjopen.bmj.com/site/about/guidelines.xhtml

What challenges did junior doctors face whilst working during the COVID-19 pandemic? A qualitative study

Abstract

Objectives: This paper reports findings exploring junior doctors' experiences of working during the COVID-19 pandemic in the UK.

Design: Qualitative study using in-depth interviews with 15 junior doctors. Interviews were audio-recorded, transcribed, anonymised and imported into NVivo 12 to facilitate data management. Data were analysed using reflexive thematic analysis.

Setting: NHS England.

Participants: A purposive sample of 12 female and three male junior doctors who indicated severe depression and/or anxiety on the DASS-21 questionnaire or high suicidality on Paykel's measure were recruited. These doctors self-identified as having lived experience of distress due to their working conditions.

Results: We report three major themes. Firstly, the challenges of working during the COVID-19 pandemic, which were both personal and organisational. Personal challenges were characterised by helplessness and included the trauma of seeing many patients dying, fears about safety and being powerless to switch off. Work-related challenges revolved around change and uncertainty, and included increasing workloads, decreasing staff numbers and negative impacts on relationships with colleagues and patients. The second theme was strategies for coping with the impact of COVID-19 on work, which were also both personal and organisational. Personal coping strategies, which appeared limited in their usefulness, were problem and emotion-focused. Several participants appeared to have moved from coping towards learned helplessness. Some organisations reacted to COVID-19 collaboratively and flexibly. Thirdly, participants reported a positive impact of the COVID-19

For peer review only - http://bmjopen.bmj.com/site/about/guidelines.xhtml

pandemic on working practices, which included simplified new ways of working – such as consistent teams and longer rotations – as well as increased camaraderie and support.

Conclusions: The trauma that junior doctors experienced whilst working during Covid-19 led to powerlessness and a reduction in the benefit of individual coping strategies. This may have resulted in feelings of resignation. We recommend that, post-pandemic, junior doctors are offered stronger psychological and practical support.

Article summary: Strengths and limitations

- Participants were interviewed at the peak of the second wave of COVID-19 during the UK, meaning transcripts contain data that are highly relevant to the research question
- In-depth, reflexive thematic analysis was carried out on the data, leading to the development of rich, insightful themes
- Female participants outnumbered male participants in this study, potentially leading to gender imbalance
- Additionally, the wider study was not initially designed to explore experiences of working during COVID-19. Instead, participants naturally discussed this topic during interviews.

Funding statement

The study was funded by NIHR Research for Patient Benefit (grant number PB-PG-0418-20023). Please note that CCG is part-funded by West Midlands ARC.

Competing interests

None

For peer review only

Introduction

Doctors are more vulnerable to mental illnesses [such as anxiety and depression] and suicide than the general population [1, 2]. In recent years, including those before the COVID-19 pandemic, UK doctors have reported understaffing, stretched resources, increased workload and burnout [3-7].

There is an additional need to attend to frontline workers' wellbeing during health crises [6-8]. Frontline workers caring for COVID-19 patients have reported stress and distress due to the strain on healthcare systems [9]. Such stressors include the need for rapid training around treating a new illness [9] and the psychological impact of exposure to unprecedented levels of suffering and COVID-19-related deaths, both of patients and colleagues [8, 10, 11].

These stressors led to healthcare professionals (HCP) reporting fears about contracting or spreading the virus as well as uncertainty due to new ways of working [11, 12]. Impacts of these fears and stressors include reduced sleep, self-harm, panic attacks, guilt, relationship breakdowns [11], concerns about lack of training [7] and psychological trauma [10].

The UK reported some of the highest numbers of COVID-19 cases in Europe [7]. In a recent paper, almost half of the 224 UK doctors surveyed (from junior doctors to consultants) felt that their mental health had been harmed by the pandemic, while a third reported impacts to their physical health [5]. Increased healthcare worker burnout is, therefore, a major concern at this time. We need a holistic understanding of the experiences and needs of frontline workers to mitigate psychological distress and burnout [11].

'Junior doctor' is the term given to qualified doctors who are still in training whilst working. They may have eight or more years of experience, depending on their speciality [13]. Junior doctors have reported fears that they will 'fail' or appear 'weak' if they take time off sick, making it harder for them to report mental health concerns [14]. This group faced unique challenges during COVID-19 due to uncertainties about exams [6], potential redeployment [8, 15, 16] and concerns about their learning opportunities [15, 16]. UK junior doctors have

For peer review only - http://bmjopen.bmj.com/site/about/guidelines.xhtml

reported that they did not receive enough education before treating COVID-19 patients [15]. They were also often faced with the difficult task of contacting patients' families to provide updates, since relatives were typically not permitted to visit [15].

Despite this, few researchers have looked in-depth at the psychological experiences of junior doctors. Instead, they have explored practical matters relating to this group, such as the resilience of new rotas (that is, assigning enough staff to cope with the workload) [17], redeployment [15, 16], the impact on training [18] and the provision of certain services such as obs and gynae [19].

Researchers have posited the need for more in-depth qualitative analysis in this area [5, 11]. This paper is part of a wider study [20, 21] designed to explore the impact of working conditions and cultures on junior doctors in general. Data collection coincided with the second wave of the pandemic in the UK, meaning the topic naturally arose for all 15 participants interviewed. As such, we aim to address this crucial gap in the literature and reflect the experiences of junior doctors working within the context of COVID-19.

Method

Study design and setting

This qualitative study is part of a larger mixed-methods study exploring junior doctors' perceptions of stress and distress. Semi-structured interviews were used to explore junior doctors' experiences of working during COVID-19. The study setting was the NHS in England.

Sampling and recruitment

A total of 456 junior doctors were initially recruited for an online survey exploring working cultures, psychological distress and suicidality between November 2020 and March 2021. They self-identified as participants, accessing the survey through posts on social media, junior doctor forums and via emails sent from their speciality schools. Survey participants whose results indicated severe depression and/or anxiety on the DASS-21 questionnaire [22] or high suicidality on Paykel's measure [23] were contacted via email to ask if they would like to take part in an in-depth, qualitative survey. As such, it should be noted that, in

For peer review only - http://bmjopen.bmj.com/site/about/guidelines.xhtml

line with our qualitative methodology, this was a small, purposive sample of junior doctors who were experiencing stress and distress as a result of their working conditions. Thus, findings cannot be generalised to all junior doctors. However, it should also be noted that levels of distress were high in the whole sample of surveyed junior doctors. A total of 27 potential participants were contacted, of which 15 were female, nine male, three undisclosed.

Interested individuals contacted JS and gave informed consent. Participants were given the chance to ask JS questions about the research team and the study before interviews went ahead. Fifteen junior doctors (12 female, three male) were recruited.

Data collection

A semi-structured interview guide was developed by the research team and modified iteratively as data collection and analysis progressed. This guide aimed to capture participants' views, experiences, feelings and beliefs about working conditions and cultures which were perceived as stressful or distressing. The guide was informed by the existing literature [1, 3, 14], input from junior doctors on the study team as well as patient and public involvement (PPI) consultation exercises conducted before obtaining funding. Following conventions for semi-structured interviews [24], points from the topic guide were followed up with individualised questions exploring topics of interest and importance for each participant.

Interviews were conducted either on the telephone or via video call, from participants' homes or places of work. They took place between December 2020 and February 2021 – that is, during the second wave of the COVID-19 pandemic in the UK – and at a date and time that were convenient to the participants. A risk protocol was used to ensure that appropriate support from two senior GPs who were on the study team and/or Practitioner Health would be provided to participants in the event of the disclosure of suicidal ideation. The in-depth interviews were conducted by JS, a female PhD psychologist with extensive qualitative methods expertise. JS also recorded any pertinent observations in field notes following each interview. Interviews ranged from 29 minutes to 102 minutes in length (mean = 62.8 minutes).

For peer review only - http://bmjopen.bmj.com/site/about/guidelines.xhtml

For peer review only

The audio-recorded interviews were transcribed verbatim and checked for accuracy by JS before analysis. All transcripts were anonymised before discussion within the wider research team. Reflexive notes were recorded by researchers throughout the process.

Patient and public involvement and engagement

There are three junior doctors on the research team, all of whom consulted with other colleagues in the PPI team about the initial research idea and participated in analysis meetings. Five junior doctors gave feedback on the initial funding application, while four fed back on the protocol, topic guide and participant-facing documents.

Data analysis

Data were analysed by JS using reflexive thematic analysis [25, 26], in which themes highlight patterns of shared meaning united by a core concept. An inductive, explicit, critical realist approach was adopted, since this was in line with the researchers' desire for a rich, data-driven analysis which demonstrated the interplay between events and participants' interpretations of those events [25]. Data saturation is not a relevant concept within this type of approach, in which it is accepted that each new participant adds fresh insights. Analysis began once all interviews had been conducted. Transcripts were analysed one by one using NVivo 12. As analysis progressed, a table of themes was generated and refined. Each new transcript led to new codes and themes being added or expanded. In addition, four members of the team, one of whom was a junior doctor and two of whom were academic GPs (RR, MB, AT, CCG), read and fed back on six of the 15 interviews. Their views and insights were collaboratively incorporated into the NVivo codes. JS then refined these codes to create relevant tables of themes once all interviews had been analysed and discussed. Analysis continued and deepened during the write-up, where shared meanings were generated and described for each theme [26].

Reflexivity

RR, the study PI, is epistemologically steeped in qualitative traditions underpinned by interpretivism and phenomenology, and is oriented by critical theory such as feminism. This

For peer review only - http://bmjopen.bmj.com/site/about/guidelines.xhtml

is likely to have influenced her interest in exploring why female doctors are more likely to experience distress.

JS, the lead analyst on this paper, is a qualitative health psychologist. She has an interest in in-depth, interpretative methods. She is white, cis-gendered, heterosexual and able-bodied. This heteronormative positioning is likely to have impacted her interviewing and analysis.

Both researchers have an interest in the systemic issues impacting individual NHS workers and are motivated by trying to find organisational – rather than individual – solutions for those workers.

The junior doctor (AT) and academic GPs (MB and CCG) who also contributed to analysis of the data have experienced and observed events during their professional lives which may have influenced how strongly they interpreted the data. Additionally, MB and CCG have a strong interest in mental health.

Findings

All fifteen participants discussed the impact of COVID-19 on their working conditions. Findings divided into three major themes: Challenges of working during the COVID-19 pandemic; Strategies for coping with the impact of COVID-19 on work; Positive impact of the COVID-19 pandemic on working practices.

See Table One for an overview of all relevant themes and subthemes.

For peer review only

Theme	Subtheme
Challenges of working during the Covid-19 pandemic<br>*"patients were just dying in front of us so quickly and they were young" (P5)*	Personal impact: Helplessness in the face of trauma<br>*"my sleep is awful again, I'm waking up, I think COVID hasn't helped" (P1)*
	Work-related impact: Change and uncertainty<br>*"I gained 14 new patients who I'd not met before" (P6)*
Strategies for coping with the impact of Covid-19 on work<br>*"So although I should have moved on from GP I ended up staying in GP so I was actually there for eight months." (P7)*	Limitations of personal strategies<br>*"I cried a lot" (P14)*
	Organisational strategies<br>*"we'd kind of share what we've learned" (P5)*
Positive impact of Covid-19 on working practices<br>*"since COVID, things have improved slightly there's, um we have something called like the rest and recuperation hub." (P6)*	Positive new ways of working<br>*"We almost looked forward to going to work" (P8)*
	Additional support and camaraderie<br>*"they provided hot meals" (P5)*

Table one: Table of themes for junior doctors' experience of working during the Covid-19 pandemic

For peer review only

Challenges of working during the COVID-19 pandemic

Participants described challenges related to their work as junior doctors during the COVID-19 pandemic. Challenges were personal or work-related.

Personal impact: Helplessness in the face of trauma

Working as junior doctors during the COVID-19 pandemic affected participants' mental health. Throughout this theme, there is a sense that participants felt helpless and powerless as they strove to carry out their jobs in such unmapped, traumatic territories.

One participant described the harmful impact of being exposed to death and suffering:

> *I'd seen [pause] a whole ward just emptied out and then refilled overnight, after people had just died. It was horrendous. Uh, I was like, "I need to talk to somebody about this or I'm just going to go home and cry". (P5, female)*

This participant's language – 'emptied out' and 'refilled' – suggests that the COVID-19 patients had become dehumanised for her; a mass of unwell bodies who were dying and being replaced in an almost mechanical manner. She was helpless to stop this flow of nameless bodies.

Participants felt helpless in the face of fears for their own safety and that of their loved ones. Initially, they were unsure of how to protect themselves or of the risk they might pose to their families:

> *…we had someone that we thought was, um, COVID, but it was very, very early on. And I remember being told off for wearing a mask. (P3, female)*

> *…we were worried about if we were taking home our clothes, if we were making other people sick, if we would get sick, it was an incredibly stressful working environment. (P5, female)*

For peer review only

As time went on, fears for personal safety came from different sources, with one participant reporting that her colleagues were not maintaining safety standards. However, as a junior doctor, she felt powerless to ask for this to change:

It's not patients, it's staff. I find that really stressful. Like you walk past an office and there might be two or three people sat in an office having a chat, all with their masks under their chin. […] I don't feel confident enough to knock on the window and be like guys, what are you doing? But I know that them doing it puts me more at risk and puts the patients at risk. […] You see stuff being wrong and you're like every day, like multiple times a day you're like do I say something, do I not say something? And you feel bad for not saying something. (P10, female)

This description of this discomfort could be defined as moral injury; that is, the distress that occurs when a person witnesses or carries out an act that is contrary to their values. The participant felt uncomfortable and helpless however she responded.

Participants felt powerless to switch off or rest when they got home from work:

You couldn't switch off, you never felt like you'd had, uh, done a good job. (P5, female)

…my sleep is awful again, I'm waking up, I think COVID hasn't helped with these sort of flashbacks. (P1, female)

Participants often did not feel supported in the new working environment caused by COVID-19, which led to further helplessness, fear and trauma. The lack of support could be practical:

I'm going to personally take responsibility for changing […] the big scary machine that I'm not trained in, and, uh, figure out how it works, whilst the patient is there trying to physically die in front of me, but so are five others, so oh well, no help is coming. (P5, female)

For peer review only - http://bmjopen.bmj.com/site/about/guidelines.xhtml

Some felt unsupported psychologically, with one participant appearing to feel that her needs were invisible to those who might support her:

> *…they got some psychologists who would be available and very occasionally they would come on the ward. [pause] And they would talk to the nurses. And that was it. No. It felt assumed to be on the nurses and people working in ITU and just ordinary junior doctors didn't [pause] didn't seem to matter. (P14, female)*

Another felt unsupported in terms of her physical health; her safety was compromised, meaning she was unable to protect herself:

> *…you will turn up on a ward and you will find out halfway through handover that they've had a positive case over the weekend. (P10, female)*

Additionally, a junior doctor whose family were overseas reported feeling unsupported by her hospital after contracting COVID-19:

> *…when I went to quarantine, I realised that no-one actually cares about you from the hospital? […] No-one called me! […] When I was very very sick, imagine that, if I had, if I had literally no-one. (P4, female)*

Work-related impact: Change and uncertainty

The work-related impact of working during a COVID-19 context centred around uncertainty and change. These included changes to workload, staffing levels, relationships with colleagues and patients, lack of support and uncertainty around new ways of working.

The junior doctors' workload grew significantly when COVID-19 hit, leading to further stressors. This led to a huge and stressful increase in one participant's responsibilities:

For peer review only - http://bmjopen.bmj.com/site/about/guidelines.xhtml

…on a Friday in the middle of the day when there was no consultant around […] I gained 14 new patients who I'd not met before […] that was a really stressful day. (P6, female)

Workload increased out of hours as well, as participants were constantly having to learn new facts about the virus and its management. The quote below demonstrates the doubt and pressure felt whilst trying to learn in the face of unmanageable amounts of new information:

So we were getting 20 emails a day, and every single one would have a red flag saying "vital, important, must read", and you'd worry you'd missed something […] there's so much information, it was constant, and you couldn't switch off, because it would impact your job. (P5, female)

As workload rose, staffing levels, which had already been stretched, were further adversely affected by further staff reductions due to illness or the need to self-isolate, demonstrating further change and uncertainty:

So it's very very short-staffed because a lot of the people are self-isolating, ill with COVID, or just because you know they've worked already five or six days in a row, and obviously they're quite tired and they have to take a break. (P12, male)

The additional workload changed working relationships in various ways. Participants reported that colleagues became irritable or verbally aggressive due to increased stress:

I think everyone got a little bit more [pause], um, maybe snippy? With each other? 'Cause we were all are very stressed and anxious. (P3, female)

For peer review only - http://bmjopen.bmj.com/site/about/guidelines.xhtml

…a registrar wearing an MF53 mask[1] and the consultant laying into him basically shouting at him that […] he was depriving someone else who actually needed this mask […] emotions were running high because people were scared. (P14, female)

One trainee, based in general practice, reported that patients had become abusive during telephone appointments, potentially dehumanising their doctors:

…sometimes people lose sense of the fact that it's another human being on the end of the phone with them. And you're already dehumanised a little bit as a doctor because people expect you to be more than, more than human. And when you then couple that with someone just being this kind of like faceless voice on the end of the phone, especially when people are scared or something like that, it just there's that heightened level of aggression. (P7, female)

That participant also reported finding the change to telephone appointments clinically challenging and risky in terms of being able to diagnose patients accurately.

I don't think you realise how much you rely on seeing someone in front of you to know how well they are. And talking to someone over the phone it just feels a lot more dangerous. (P7, female)

Compounding these changes which made participants' working lives harder was the fact that it also became harder to speak with and get support from peers due to the safety measures:

Um, and now with COVID where you're only allowed, like, two people in each room, it, it's very difficult to, um, socialise and talk. (P8, female)

As junior doctors in training, participants also found the uncertainty around changes to rotas and exams challenging:

[1] This is a full face, military style of mask

For peer review only

> *…a fair amount of uncertainty and the problem this time is that, ah, a lot of courses are still going ahead, exams are still going ahead, but we've been moved onto emergency rotas with a week's notice. (P8, female)*

The junior doctors were often expected to work in different specialities or locations from those which they had been allocated to pre-pandemic. Constant anxiety due to uncertainty about redeployment was reported:

> *…anxious and uncertain about whether that was going to happen and would sort of check my emails pretty consistently to see whether that was actually whether that was going to be um delayed or stopped because of COVID redeployment. (P6, female)*

The pandemic meant that new ways of working were quickly developed and implemented. Trying to adjust to these changes was another challenge. One trainee in psychiatry talked about the potential stress and impact on patient care of working from home:

> *…you've not got those people around you to bounce things off, so you might get an email and it might be quite an anxiety-inducing email because it might say someone's suicidal, you need to see them, and you're thinking, oh, I can't see them, erm, and normally, kind of in an office you'd just be able to ask, can anyone else see them? (P2, female)*

Strategies for coping with the impact of COVID-19 on work

Participants described both personal and organisational strategies for coping with the above challenges.

For peer review only - http://bmjopen.bmj.com/site/about/guidelines.xhtml

Limitations of personal coping strategies

Emotion-focused and problem-focused coping strategies were utilised for dealing with the challenges of COVID-19. However, there was a sense that these personal coping strategies, which might have been adequate before COVID-19, were not enough to protect participants from the impact of working during the pandemic.

Emotion-based coping strategies included crying:

> *So I cried a lot outside. Because it was getting warmer so you could go outside. Hug a tree, cry. (P14, female)*

Stoicism was used by some, although this latter strategy suggested a sense of resignation, illustrated by P8's rhetorical question:

> *And [pause] and in a way it didn't really matter that our rota changed, because there was nothing else to do? (P8, female)*

A sense of powerlessness combined with acceptance was perceived to have impacted the profession as a whole:

> *I think erm you know everyone's a bit more sort of resigned to things now and it feels like we've sort of erm entered a collective sort of depressive state of acceptance. (P9, male)*

We can see that these personal, emotion-based coping strategies had their limits when employed during the COVID-19 pandemic.

For peer review only - http://bmjopen.bmj.com/site/about/guidelines.xhtml

Problem-focused strategies were perhaps more effective. One participant volunteered to take on the work of calling relatives to let them know their loved ones were very sick, perhaps to be able to provide a more personal input to such a traumatic situation.

> *I used to volunteer to kind of be the person making those phone calls, cos it was, it felt like you were able to do something about it at least? It wasn't that sort of like, "I put lines in people and hopefully", and then just watching them die. (P5, female)*

Another participant agentically took control of her situation by arranging more support for herself, perhaps in response to the helplessness described in the previous theme:

> *…when lockdown came back in […] I noticed that like I was feeling low so I referred myself to the Let's Talk Wellbeing, erm which is like the community, CBT, GP, self-referral system. And I found that really helpful erm so that kind of stopped me spiralling. (P10, female)*

Organisational strategies

Just as participants found ways to cope with the challenges of COVID-19, so did the organisations and teams for whom they worked, with some trusts and teams demonstrating collaborative, flexible thinking. One participant reported flexibility in terms of working from home for colleagues who had to self-isolate:

> *…most of the places have let the person sort of choose whether they you know, if it's your child that's got a fever and actually you know you're isolating and could do things then that's fine. But if you're poorly then you're poorly and that's fine. (P11, female)*

Another described the need for her team to make pragmatic decisions about how to treat COVID-19 patients:

For peer review only - http://bmjopen.bmj.com/site/about/guidelines.xhtml

For peer review only

So if someone was clearly dying, they would [pause] be stepped down to a normal ward because on a normal ward they could at least have a visitor for one hour a day. (P14, female)

A third participant reported that her team pulled together to help one another in the new circumstances:

…there'd be so many [emails] even coming in during our shift, we'd divide it up, so we'd say, "you read these five, I'll read these five, you read these five, and then I'll read these five", and then we'd kind of share what we've learned from them. (P5, female)

Positive impact of COVID-19 on working practices

Participants reported that working as junior doctors during the COVID-19 pandemic had some positive impacts. These were included new and less bureaucratic ways of working as well as additional support and camaraderie.

Positive new ways of working

Positive changes revolved around a less bureaucratic way of working, which included consistent teams, longer rotations and less red tape.

Several participants reported that they were now working in a consistent team, rather than regularly working with new colleagues. This was experienced as positive:

So normally, you're kind of working with somebody new every day almost. But we worked in teams that didn't rotate, so you had […] this team that you worked with very intensely for those four months as well, and that support structure was really good. (P5, female)

For peer review only - http://bmjopen.bmj.com/site/about/guidelines.xhtml

…we got really to know each other, we had a little social WhatsApp group where we'd, like, post pictures of the cakes we were gonna bring in, you know, everyone bought in food. We almost looked forward to going to work because you were like, oh, my buddies are there. (P8, female)

A sense of being part of a team who enjoy work comes across in the above quote, where cake and conversation bring some positivity to bleak picture painted thus far.

Rotations were paused for many junior doctors. Although this could lead to uncertainty, as reported in an earlier theme, it also had some potentially positive impacts:

So we were on the first rotation for four months and then the second for eight months […] Um, so, I guess it would have depended on what ward you got stuck on? [interviewer laughs] Um, I got stuck on one of the nice placements, I really enjoyed myself on the ward. (P3, female)

Various practical changes to working patterns were also experienced as positive. These included simple factors such as the ability to work from home and reduced red tape:

…just get away and do something relaxing, even if it's just go for a walk around the local canal and come back on a lunchtime is so much more achievable when you're working from home. So I think that's been really good. (P2, female)

…they say oh, we want you to travel to a hospital on your day off to show us your passport and your GMC certificate. And it's like I've been – doing this for 10 years. I've worked for you six times! Like, you've got my details [both laugh]. And that's one thing where COVID has been really good because now they do it online and I'm like, why couldn't you have always done this? (P8, female)

One participant even appeared to cite COVID-19 as a motivator for returning to work at the NHS after time in another career:

For peer review only - http://bmjopen.bmj.com/site/about/guidelines.xhtml

> *...then COVID came and I wanted to come back to medicine anyway so I thought okay fine then let's just crack on with erm with the NHS. (P15, female)*

Additional support and camaraderie

Some participants reported that new supportive measures, such as additional facilities, had been put in place by their workplaces:

> *...they provided hot meals, which, at the beginning, when there were huge queues at the supermarket, and we were working 12-hour shifts, five days a week, and, um, [pause] and it was unpredictable whether you could kind of get food, because there were a lot of shortages and things. (P5, female)*

> *And since COVID, things have improved slightly, there's, um we have something called like the rest and recuperation hub, which is like a room erm that does free teas and coffees and a few snacks [...] you go there on your breaks to relax. (P6, female)*

Another participant reported that her hospital made an effort to offer junior doctors support, although this was against the backdrop of a toxic working environment:

> *I'd say the culture's getting worse except for the fact that they send an e-mail out every now and again with some contact numbers [for support services] and that's what COVID has done. (P1, female)*

It should be noted that the reports of improved support were tempered – as in the description of 'slight' improvements to a culture that was also described as 'getting worse'.

Discussion

Fifteen distressed junior doctors were interviewed between December 2020 and February 2021 about their perceptions of stress and distress in their workplace cultures. All

For peer review only

participants discussed how COVID-19 impacted their experiences. Looking at our themes as a gestalt, we suggest that the helplessness that arose due to the trauma of working during the pandemic meant that individual coping strategies which may have been more beneficial during less unusual times fell short, something that often went unrecognised by employers. To compound this, participants were also not sufficiently supported either practically or psychologically during this time. This may have led to feeling powerless and resigned in the face of difficult circumstances for which they were unprepared. Additionally, we recommend that the positive lessons highlighted in this paper are adhered to.

Helplessness was commonly reported whilst during in the context of COVID-19. Specifically, one participant described how traumatic it was to see so many patients dying. Others [8, 10, 27] have cited grief and managing such large numbers of patient deaths as especially challenging. We suggest that newer junior doctors might need extra support to process grief in such exceptional circumstances, for which they had not been trained. This might especially be the case for younger doctors [28] and female doctors [29, 1] since it has been shown that these groups, who made up the majority of participants in the current study, are potentially more vulnerable to depression, stress and suicidal thoughts.

Another participant reported an experience of moral injury following the unsafe behaviour of other staff members. Moral injury due to redeployment away from long-term patients [15] and concerns about letting patients down [8] during COVID-19 has also been reported. Our findings add an additional perspective, demonstrating that moral injury can also arise due to staff members neglecting safety protocols.

Adding to these traumatic personal experiences, participants reported that while their workload rose due, staffing levels often decreased. Previous research has shown that, following austerity [30], UK HCPs were already working in an under-resourced environment and that additional workload is a potent stressor [21, 31-33]. Crises such as COVID-19 further emphasise the need for extra resources for our healthcare system, echoing the recommendations of the 2009 Boorman report [34], which have been widely neglected [6]. It is often harder for frontline workers to take breaks during a pandemic [5, 10], adding to the potential for burnout since longer working hours are a risk factor [29]. Cubitt and

For peer review only - http://bmjopen.bmj.com/site/about/guidelines.xhtml

colleagues [5] have highlighted the need for rotas that enable well-being rather than merely being resilient. Qualitative research such as the current study adds depth to these recommendations by demonstrating the instability, lack of support and powerlessness that distressed HCPs faced during this time.

Participants felt unsupported whilst working in these new, traumatic circumstances, a finding reflected elsewhere [7, 10, 11]. For example, one participant who needed psychological support intimated that she felt invisible. Whilst the needs of others – nurses and non-medical staff – were considered, her needs were assumed not to exist, demonstrating the powerlessness of the junior doctors in this situation. If you cannot be seen, you cannot be helped.

The need for extra training and support for junior doctors during pandemics has been reported [15]. We echo this recommendation and would add that support could come from good leadership which recognises the challenges staff face, a feeling of being valued within a team and by addressing the practical and physical limitations junior doctors frequently experience, such as poor 'on call' accommodation and access to regular meals. We suggest that employers often fail to recognise the limitations of individual coping strategies, both during crises such as the pandemic and in less unusual times.

Participants used various strategies to attempt to cope with working during COVID-19. Emotion-focused strategies such as crying were reported in our study, although these strategies often appeared limited in usefulness. At times, the stoicism reported by participants in the current study verged on learned helplessness, demonstrating that personal coping strategies alone are not enough, and that coping is not guaranteed in a healthcare crisis when doctors are already stressed and distressed. Various individualised coping strategies have been suggested, including healthy eating, attending training, going to therapy, support networks [6] and making use of 'wobble rooms' [15]. However, San Juan and colleagues [6] recognised that finding time for these activities might be difficult, particularly during a time of crisis.

For peer review only - http://bmjopen.bmj.com/site/about/guidelines.xhtml

For peer review only

Owens et al. [30] state that if we are continually asking our HCPs to behave heroically in exceptional circumstances, we are inviting burnout. Indeed, it could be posited that encouraging such strategies places the responsibility for managing the unmanageable with individuals, rather than the system [6]. It is argued that, in our current neo-liberal culture, responsibility for wellbeing is often placed on the individual, exonerating the state and systems for the wellbeing of workers [30, 35, 36]. This can be seen in the use of the term 'resilience', which places responsibility for managing the unmanageable on the shoulders of individuals, rather than organisations [35, 36]. Therefore, in line with San Juan et al. [6], we recommend a focus on organisational, rather than personal, coping strategies. Those organisational strategies should, as in our findings, include flexibility and better organisational, managerial and peer-support through teamwork and collaboration as well as addressing the practical workplace issues which could lead to HCPs feeling physically safe and cared for. Vulnerable junior doctors need organisational support especially, although not exclusively, during crises like COVID-19. However, the emphasis continues to be on individual [37, 38].

Participants reported several potentially positive impacts of working during the pandemic, a novel finding. These included working in more consistent teams. San Juan and colleagues [6] have similarly reported that consistent teams are helpful for HCPs, while inconsistent teams make it harder for junior doctors to seek support [14], increasing stress and vulnerability to mental ill-health [21]. As such, we recommend that, where possible, policymakers consider the use of consistent teams for junior doctors going forward. The beneficial impact of a reduction in bureaucracy reported by one participant appears to be another novel finding. We would suggest any such reductions should be maintained after the pandemic ends, with a potential reduction in time pressures for junior – and senior – doctors.

Participants stated that some new supportive measures, such as rest hubs, had been put into place during COVID-19. Such spaces have been deemed helpful by other researchers [7, 10, 15], although there are anecdotal reports that many of these spaces have now been closed as hospitalisations from COVID-19 reduce. In contrast, HCPs in other studies have

For peer review only - http://bmjopen.bmj.com/site/about/guidelines.xhtml

reported that the extra strain on the system meant that there were fewer places than usual to shower, rest or relax with colleagues [5, 6].

In line with our findings, Vindrola-Padros et al. [7] reported that there was extra signposting towards support during COVID-19; however, there was not often time to engage with this support. Additionally, it has been anecdotally reported that much of this support has been withdrawn now. This adds further weight to the notion that systemic, holistic changes are needed to support NHS staff, rather than focusing the responsibility for change on individuals [6]. We suggest that such limited responses from employers may have contributed to the feelings of resignation described by some of our participants.

Limitations

This study has various strengths, including being the first qualitative paper (to our knowledge) to explore the experiences of junior doctors during COVID-19. Our data was collected during the pandemic and we utilised in-depth, collaborative thematic analysis. However, despite these strengths, the paper has several limitations. We did not recruit these participants specifically to talk about the Covid-19 pandemic. Rather, the timing of the study meant that the topic arose naturally. As such, the interview guide could have been designed to ask participants more thoroughly about these experiences. Additionally, some of the junior doctors had more experience of working with COVID-19 patients than others, meaning some participants are better represented in this paper than others. Further, there is a notable gender disparity, with a higher proportion of female doctors taking part. More female (n=12) participants volunteered than males (n=3). The increased willingness of female participants to speak about their experiences may be associated with evidence indicating that female doctors are more likely to experience distress. Sadly, this group are also more likely to kill themselves [1]. The higher proportion of female participants may also reflect gendered help-seeking behaviour for mental ill-health, evidenced in the wider population [39], as well as the fact that female doctors are more likely to take part in research than their male counterparts [40]. Finally, it should be reiterated that this was a purposive sample of particularly distressed junior doctors, albeit taken from a wider sample

For peer review only

in which distress was widely reported, and so our findings are not intended to be generalised to all junior doctors.

Conclusions and recommendations

We conclude that junior doctors working during the COVID-19 pandemic faced multiple stressors and used various coping mechanisms to deal with these, with greater or lesser degrees of success. Several unexpected benefits of this period arose, including new ways of working and additional support and camaraderie. We believe that the responsibility for alleviating the stress and distress of junior doctors working during times of stress lies with organisational employment issues and systemic workforce gaps, rather than with individuals. As such, we recommend system-wide changes, such as improved communication strategies, increased flexibility around home-based working, addressing the physical limitations of the working conditions many junior doctors experience and more supportive and compassionate leadership. Additionally, we suggest that, where possible, junior doctors are assigned to consistent teams, with the opportunity for appropriate psychological support where indicated.

Author contributions: RR, JS, CCG, MB, AD, KT, AG, AT, MvH, LA, JM – substantial contributions to conception and design; approval of final version to the published.

RR, JS, CCG, MB, AT, KT, AD, AT – acquisition, analysis or interpretation of data; drafting the article or revising it critically for important intellectual content.

Acknowledgements: The authors would like to thank all junior doctor participants who kindly gave their time to share their experiences of working in the NHS. We also wish to thank members of the PPIE group who provided valuable input throughout the study.

Data sharing: This study has not received ethical approval to share confidential data with any third party other than the research team.

For peer review only

Ethical approval: Ethical approval was granted by the University of Birmingham and Health Research Authority (reference number: 19/HRA/6579)

For peer review only

For peer review only - http://bmjopen.bmj.com/site/about/guidelines.xhtml

References

1. Dutheil, F., et al. *Suicide among physicians and health-care workers: A systematic review and meta-analysis*. PloS one. 2019. **14**(12): p. e0226361

2. Petrie, K., et al. *Interventions to reduce symptoms of common mental disorders and suicidal ideation in physicians: A systematic review and meta-analysis*. The Lancet Psychiatry. 2019. **6**(3): 225-34.

3. Brooks, S. K., et al. *Review of literature on the mental health of doctors: Are specialist services needed?* Journal of Mental Health. 2011. **20**(2): 146-56.

4. Chopra, S. S. et al. *Physician Burnout*. JAMA. 2004. **291**(5): 633–33.

5. Cubitt, L. J. et al. *Beyond PPE: A mixed qualitative-quantitative study capturing the wider issues affecting doctors' well-being during the COVID-19 pandemic*. BMJ Open. 2021. **11**(3): e050223.

6. San Juan, N. V., et al. *Healthcare Workers' mental health and wellbeing during the COVID-19 pandemic in the UK: Contrasting guidelines with experiences in practice*. MedRxiv. 2020; advanced online.

7. Vindrola-Padros C., et al. *Perceptions and experiences of healthcare workers during the COVID-19 pandemic in the UK*. BMJ Open. 2020; **10**(11): Advanced online: e040503.

8. Liberati E., et al. *A qualitative study of experiences of NHS mental healthcare workers during the Covid-19 pandemic*. BMC Psychiatry. 2021. **21**(1): 1-12.

9. Sun, N., et al. *A qualitative study on the psychological experience of caregivers of COVID-19 patients.* American Journal of Infection Control. 2020. **48**(6): 592-8.

10. Blake, H., et al. *COVID-well study: Qualitative evaluation of supported wellbeing centres and psychological first aid for healthcare workers during the COVID-19 pandemic.* International Journal of Environmental Research and Public Health. 2021. **18**(7): 3626-59.

11. Newman K. L., et al. *Experiences and emotional strain of NHS frontline workers during the peak of the COVID-19 pandemic*. International Journal of Social Psychiatry. 2021; advanced online 00207640211006153.

12. Cawcutt K. A., et al. *Fighting fear in healthcare workers during the COVID-19 pandemic*. Infection Control & Hospital Epidemiology. 2020. **41**(10): 1192-3.

13. BMA, *https://www.bma.org.uk/advice-and-support/international-doctors/life-and-work-in-the-uk/toolkit-for-doctors-new-to-the-uk/doctors-titles-explained.* 2020.

For peer review only

14. Fox F. E., et al. *Junior doctors' experiences of personal illness: A qualitative study*. Medical Education. 2011. **45**(12): 1251-61.

15. Coughlan C., et al. *COVID-19: Lessons for junior doctors redeployed to critical care*. Postgraduate Medical Journal. 2021. **97**(1145): 188-91.

16. Lion P., et al. *ENT trainees' experience of redeployment during the coronavirus disease 2019 pandemic: A qualitative study*. The Journal of Laryngology & Otology, 2021. 1-13.

17. Aziz, A., et al. *Rapid transformation of the medical on-call rotas to deal with the COVID-19 pandemic: A case study*. Future Healthcare Journal. 2021; **8**(1): e36.

18. Seifman M. A., et al. *COVID-19 impact on junior doctor education and training: A scoping review*. Postgraduate medical journal. 2021.

19. Rimmer, M. P., et al. *Provision of obstetrics and gynaecology services during the COVID-19 pandemic: A survey of junior doctors in the UK National Health Service*. BJOG: An International Journal of Obstetrics & Gynaecology. 2020. **127**(9): 1123-8.

20. Riley, R., et al. *Protective factors and sources of support in the workplace as experienced by UK foundation and junior doctors: A qualitative study*. BMJ Open. 2021. **11**(6): e045588.

21. Riley, R., et al. *Sources of work-related psychological distress experienced by UK-wide foundation and junior doctors: A qualitative study*. BMJ Open. 2021. **11**(6): e043521.

22. Osman, A., et al. *The depression anxiety stress scales—21 [DASS-21]: Further examination of dimensions, scale reliability, and correlates*. Journal of clinical psychology. 2012. **68**(12): 1322–1338.

23. Paykel, E. S., et al. *Suicidal feelings in the general population: A prevalence study.* The British Journal of Psychiatry. 1974. **214**(582): 460–9.

24. King N. and C. Horrocks, *Interviews in Qualitative Research*. 2010, London: Sage.

25. Braun V. and V. Clarke, *Using thematic analysis in psychology*. Qualitative research in psychology, 2006. **3**(2): 77-101.

26. Braun V. and V. Clarke, *Reflecting on reflexive thematic analysis*. Qualitative Research in Sport, Exercise and Health, 2019. **11**(4): 589-97.

27. Johnson, S., et al. *Impact on mental health care and on mental health service users of the COVID-19 pandemic: A mixed methods survey of UK mental health care staff*. Social Psychiatry and Psychiatric Epidemiology, 2021. **56**(1): 25-37.

For peer review only - http://bmjopen.bmj.com/site/about/guidelines.xhtml

For peer review only

28. Tallentire VR, Smith SE, Facey AD, Rotstein L. Exploring newly qualified doctors' workplace stressors: an interview study from Australia. BMJ Open. 2017;7(8):e015890.

29. Amoafo, E., et al. *What are the significant factors associated with burnout in doctors?* Occupational Medicine, 2015; **65**(2): 117-21.

30. Owens, J., Singh, G., & Cribb, A. (2019). Austerity and professionalism: being a good healthcare professional in bad conditions. *Health Care Analysis*, *27*(3), 157-170.

31. Kinman G. and K. Teoh K, *What could make a difference to the mental health of UK doctors? A review of the research evidence*. 2018.

32. Kinman, G., et al. *The mental health and wellbeing of nurses and midwives in the United Kingdom*. Royal College of Nursing Foundation and the Society for Occupational Medicine, 2020.

33. Oliver, D., *The NHS's understaffing is its Achilles' heel*. BMJ: British Medical Journal (Online), 2017. 357.

34. Boorman, S. *NHS Health and well-being review: Interim report*. NHS Health and Wellbeing Review, 2009.

35. Joseph, J., *Resilience as embedded neoliberalism: A governmentality approach*. Resilience, 2013. **1**(1): 38-52.

36. Traynor, M. *Guest editorial: What's wrong with resilience*. Journal of Research in Nursing, 2018. **23**(1): 5-8.

37. Pollock, A., et al. *Interventions to support the resilience and mental health of frontline health and social care professionals during and after a disease outbreak, epidemic or pandemic: A mixed methods systematic review*. Cochrane Database of Systematic Reviews, 2020. 11.

38. Sood, A., et al. *Stress management and resilience training among department of medicine faculty: A pilot randomized clinical trial*. Journal of general internal medicine, 2011. **26**(8): 858-61.

39. Seidler, Z. E., et al. *The role of masculinity in men's help-seeking for depression: A systematic review*. Clinical Psychology Review, 2016. **49**: 106-18.

40. Cull, W. L., et al. *Response rates and response bias for 50 surveys of pediatricians.* Health Services Research, 2005. **40**(1): 213-26.

For peer review only - http://bmjopen.bmj.com/site/about/guidelines.xhtml

Consolidated criteria for reporting qualitative studies [COREQ]:

Developed from: Tong, A., Sainsbury, P., & Craig, J. [2007]. Consolidated criteria for reporting qualitative research [COREQ]: a 32-item checklist for interviews and focus groups. *International journal for quality in health care*, *19*[6], 349-357.

No. Item	Guide questions/description	Reported on page #
**Domain 1: Research team and reflexivity**		
Personal characteristics		
1. Interviewer/ facilitator	Which author/s conducted the interview or focus group?	Page 11
2. Credentials	What were the researcher's credentials? E.g. PhD, MD.	Page 11
3. Occupation	What was their occupation at the time of the study?	Page 11
4. Gender	Was the researcher male or female?	Page 11
5. Experience and training	What experience or training did the researcher have?	Page 11
Relationship with participants		
6. Relationship established	Was a relationship established prior to study commencement?	Page 11
7. Participant knowledge of interviewer	What did the participant know about the researcher? E.g. personal goals, reasons for doing the research.	Page 11
8. Interviewer characteristics	What characteristics were reported about the interviewer/facilitator? E.g. bias, assumptions, reasons and interests in the research topic	Page 12
**Domain 2: study design**		
Theoretical framework		
9. Methodological orientation and theory	What methodological orientation was stated to underpin the study? E.g. grounded theory, discourse analysis, ethnography, phenomenology, content analysis	Page 12

For peer review only

Participant sampling		
10. Sampling	How were participants selected? E.g. purposive, convenience, consecutive, snowball	Page 10
11. Method of approach	How were participants approached? E.g. face-to-face, telephone, mail, email	Page 10
12. Sample size	How many participants were in the study?	Page 11
13. Non-participation	How many people refused to participate or dropped out? Reasons?	None
Setting		
14. Setting of data collection	Where was the data collected? E.g. home, clinic, workplace	Page 11
15. Presence of non-participants	Was anyone else present besides the participants and the researchers?	No
16. Description of the sample	What were the important characteristics of the sample? E.g. demographic data, date	Page 11
Data collection		
17. Interview guide	Were questions, prompts, guides provided by the authors? Was it pilot tested?	Page 11
18. Repeat interviews	Were repeat interviews carried out? If yes, how many?	No
19. Audio/visual recording	Did the research use audio or visual recording to collect the data?	Page 11
20. Field notes	Were field notes made during and/or after the interview or focus group?	Page 11
21. Duration	What was the duration of the interviews or focus group?	Page 11
22. Data saturation	Was data saturation discussed?	Page 12
23. Transcripts returned	Were transcripts return to participants for comment and/or correction?	No, due to lack of resources
**Domain 3: analysis and findings**		
Data analysis		
24. Number of data coders	How many data coders coded the data?	Page 12
25. Description of the coding tree	Did authors provide a description of the coding tree?	No
26. Derivation of themes	Derived from the data?	Page 12

For peer review only

27. Software	What software, if applicable, was used to manage the data?	Page 12
28. Participant checking	Did participants provide feedback on the findings?	No, due to lack of resources
Reporting		
29. Quotations presented	Were participant quotations presented to illustrate the themes/findings? Was each quotation identified? E.g. participant number	Pages 13-22
30. Data and findings consistent	Was there consistency between the data presented and the findings	Yes, see sages 13-22
31. Clarity of major themes	Were major themes presented in the findings?	Yes
32. Clarity of minor themes	Is there a description of diverse cases or discussion of minor themes?	Yes

For peer review only

BMJ Open

What challenges did junior doctors face whilst working during the COVID-19 pandemic? A qualitative study

Journal:	*BMJ Open*
Manuscript ID	bmjopen-2021-056122.R2
Article Type:	Original research
Date Submitted by the Author:	11-Nov-2021

Complete List of Authors:	Spiers, Johanna; University of Birmingham, College of Medical and Dental Sciences<br>Buszewicz, Marta; University College London, Research Department of Primary Care and Population Health<br>Chew-Graham, Carolyn; University of Keele<br>Dunning, Alice; Bradford Teaching Hospitals NHS Foundation Trust<br>Taylor, Anna; University of Leeds<br>Gopfert, Anya; Torbay Council<br>Van Hove, Maria; University of Exeter<br>Teoh, Kevin; Birkbeck University of London, Department of Organizational Psychology<br>Appleby, Louis; University of Manchester, Psychiatry & Behavioral Sciences<br>Martin, James; University of Birmingham, Institute of Applied Health Research<br>Riley, Ruth; University of Birmingham Institute of Applied Health Research; University of Birmingham, of Applied Health Research

**Primary Subject Heading**:	Mental health
Secondary Subject Heading:	Health services research, Medical education and training, Qualitative research
Keywords:	COVID-19, MENTAL HEALTH, Depression & mood disorders < PSYCHIATRY, QUALITATIVE RESEARCH

SCHOLARONE™ Manuscripts

I, the Submitting Author has the right to grant and does grant on behalf of all authors of the Work (as defined in the below author licence), an exclusive licence and/or a non-exclusive licence for contributions from authors who are: i) UK Crown employees; ii) where BMJ has agreed a CC-BY licence shall apply, and/or iii) in accordance with the terms applicable for US Federal Government officers or employees acting as part of their official duties; on a worldwide, perpetual, irrevocable, royalty-free basis to BMJ Publishing Group Ltd ("BMJ") its licensees and where the relevant Journal is co-owned by BMJ to the co-owners of the Journal, to publish the Work in this journal and any other BMJ products and to exploit all rights, as set out in our licence.

The Submitting Author accepts and understands that any supply made under these terms is made by BMJ to the Submitting Author unless you are acting as an employee on behalf of your employer or a postgraduate student of an affiliated institution which is paying any applicable article publishing charge ("APC") for Open Access articles. Where the Submitting Author wishes to make the Work available on an Open Access basis (and intends to pay the relevant APC), the terms of reuse of such Open Access shall be governed by a Creative Commons licence – details of these licences and which Creative Commons licence will apply to this Work are set out in our licence referred to above.

Other than as permitted in any relevant BMJ Author's Self Archiving Policies, I confirm this Work has not been accepted for publication elsewhere, is not being considered for publication elsewhere and does not duplicate material already published. I confirm all authors consent to publication of this Work and authorise the granting of this licence.

For peer review only

What challenges did junior doctors face whilst working during the COVID-19 pandemic? A qualitative study

Johanna Spiers (corresponding author)

Institute of Applied Health Research

University of Birmingham

Birmingham

United Kingdom

johanna.spiers@gmail.com

Tel: 07966 763 364

Marta Buszewicz

Research Department of Primary Care and Population Health

University College London Medical School

London

United Kingdom

Carolyn A. Chew-Graham

School of Medicine

Keele University

Keele

United Kingdom

Alice Dunning

Yorkshire Quality and Safety Research Group

Bradford Institute for Health research

Bradford Teaching Hospitals NHS Foundation Trust

Bradford

United Kingdom

For peer review only - http://bmjopen.bmj.com/site/about/guidelines.xhtml

For peer review only

Anna K. Taylor

Leeds Institute of Health Sciences

Faculty of Medicine and Health

University of Leeds

Leeds

United Kingdom

Anya Gopfert

Torbay Council,

Torquay,

TQ1 3DR

Maria van Hove

The University of Exeter,

Exeter,

EX4 4PY

Kevin Rui-Han Teoh

Department of Organizational Psychology

Birkbeck, University of London

London

United Kingdom

Louis Appleby

Division of Psychology and Mental Health

School of Medicine

University of Manchester

Manchester

United Kingdom

James Martin

For peer review only

Institute of Applied Health Research
University of Birmingham
Birmingham
United Kingdom

Ruth Riley
Institute of Applied Health Research
University of Birmingham
Birmingham
United Kingdom

Keywords: Junior doctors, COVID-19, Occupational Stress, Mental Health, Working Conditions, Qualitative Methods

For peer review only - http://bmjopen.bmj.com/site/about/guidelines.xhtml

What challenges did junior doctors face whilst working during the COVID-19 pandemic? A qualitative study

Abstract

Objectives: This paper reports findings exploring junior doctors' experiences of working during the COVID-19 pandemic in the UK.

Design: Qualitative study using in-depth interviews with 15 junior doctors. Interviews were audio-recorded, transcribed, anonymised and imported into NVivo 12 to facilitate data management. Data were analysed using reflexive thematic analysis.

Setting: NHS England.

Participants: A purposive sample of 12 female and three male junior doctors who indicated severe depression and/or anxiety on the DASS-21 questionnaire or high suicidality on Paykel's measure were recruited. These doctors self-identified as having lived experience of distress due to their working conditions.

Results: We report three major themes. Firstly, the challenges of working during the COVID-19 pandemic, which were both personal and organisational. Personal challenges were characterised by helplessness and included the trauma of seeing many patients dying, fears about safety and being powerless to switch off. Work-related challenges revolved around change and uncertainty, and included increasing workloads, decreasing staff numbers and negative impacts on relationships with colleagues and patients. The second theme was strategies for coping with the impact of COVID-19 on work, which were also both personal and organisational. Personal coping strategies, which appeared limited in their usefulness, were problem and emotion-focused. Several participants appeared to have moved from coping towards learned helplessness. Some organisations reacted to COVID-19 collaboratively and flexibly. Thirdly, participants reported a positive impact of the COVID-19

For peer review only - http://bmjopen.bmj.com/site/about/guidelines.xhtml

pandemic on working practices, which included simplified new ways of working – such as consistent teams and longer rotations – as well as increased camaraderie and support.

Conclusions The trauma that junior doctors experienced whilst working during Covid-19 led to powerlessness and a reduction in the benefit of individual coping strategies. This may have resulted in feelings of resignation. We recommend that, post-pandemic, junior doctors are assigned to consistent teams and offered ongoing support.

Article summary: Strengths and limitations

- Participants were interviewed at the peak of the second wave of COVID-19 during the UK, meaning transcripts contain data that are highly relevant to the research question
- In-depth, reflexive thematic analysis was carried out on the data, leading to the development of rich, insightful themes
- Female participants outnumbered male participants in this study, potentially leading to gender imbalance
- Additionally, the wider study was not initially designed to explore experiences of working during COVID-19. Instead, participants naturally discussed this topic during interviews.

Funding statement

The study was funded by NIHR Research for Patient Benefit (grant number PB-PG-0418-20023). Please note that CCG is part-funded by West Midlands ARC.

Competing interests

None

For peer review only

For peer review only - http://bmjopen.bmj.com/site/about/guidelines.xhtml

Introduction

Doctors are more vulnerable to mental illnesses [such as anxiety and depression] and suicide than the general population [1, 2]. In recent years, including those before the COVID-19 pandemic, UK doctors have reported understaffing, stretched resources, increased workload and burnout [3-7].

There is an additional need to attend to frontline workers' wellbeing during health crises [6-8]. Frontline workers caring for COVID-19 patients have reported stress and distress due to the strain on healthcare systems [9]. Such stressors include the need for rapid training around treating a new illness [9] and the psychological impact of exposure to unprecedented levels of suffering and COVID-19-related deaths, both of patients and colleagues [8, 10, 11].

These stressors led to healthcare professionals (HCP) reporting fears about contracting or spreading the virus as well as uncertainty due to new ways of working [11, 12]. Impacts of these fears and stressors include reduced sleep, self-harm, panic attacks, guilt, relationship breakdowns [11], concerns about lack of training [7] and psychological trauma [10].

The UK reported some of the highest numbers of COVID-19 cases in Europe [7]. In a recent paper, almost half of the 224 UK doctors surveyed (from junior doctors to consultants) felt that their mental health had been harmed by the pandemic, while a third reported impacts to their physical health [5]. Increased healthcare worker burnout is, therefore, a major concern at this time. We need a holistic understanding of the experiences and needs of frontline workers to mitigate psychological distress and burnout [11].

'Junior doctor' is the term given to qualified doctors who are still in training whilst working. They may have eight or more years of experience, depending on their speciality [13]. Junior doctors have reported fears that they will 'fail' or appear 'weak' if they take time off sick, making it harder for them to report mental health concerns [14]. This group faced unique challenges during COVID-19 due to uncertainties about exams [6], potential redeployment [8, 15, 16] and concerns about their learning opportunities [15, 16]. UK junior doctors have

For peer review only - http://bmjopen.bmj.com/site/about/guidelines.xhtml

reported that they did not receive enough education before treating COVID-19 patients [15]. They were also often faced with the difficult task of contacting patients' families to provide updates, since relatives were typically not permitted to visit [15].

Despite this, few researchers have looked in-depth at the psychological experiences of junior doctors. Instead, they have explored practical matters relating to this group, such as the resilience of new rotas (that is, assigning enough staff to cope with the workload) [17], redeployment [15, 16], the impact on training [18] and the provision of certain services such as obs and gynae [19].

For peer review only

Researchers have posited the need for more in-depth qualitative analysis in this area [5, 11]. This paper is part of a wider study [20, 21] designed to explore the impact of working conditions and cultures on junior doctors in general. Data collection coincided with the second wave of the pandemic in the UK, meaning the topic naturally arose for all 15 participants interviewed. As such, we aim to address this crucial gap in the literature and reflect the experiences of junior doctors working within the context of COVID-19.

Method

Study design and setting

This qualitative study is part of a larger mixed-methods study exploring junior doctors' perceptions of stress and distress. Semi-structured interviews were used to explore junior doctors' experiences of working during COVID-19. The study setting was the NHS in England.

Sampling and recruitment

A total of 456 junior doctors were initially recruited for an online survey exploring working cultures, psychological distress and suicidality between November 2020 and March 2021. They self-identified as participants, accessing the survey through posts on social media, junior doctor forums and via emails sent from their speciality schools. Survey participants whose results indicated severe depression and/or anxiety on the DASS-21 questionnaire [22] or high suicidality on Paykel's measure [23] were contacted via email to ask if they would like to take part in an in-depth, qualitative survey. As such, it should be noted that, in

For peer review only - http://bmjopen.bmj.com/site/about/guidelines.xhtml

line with our qualitative methodology, this was a small, purposive sample of junior doctors who were experiencing stress and distress as a result of their working conditions. Thus, findings cannot be generalised to all junior doctors. However, it should also be noted that levels of distress were high in the whole sample of surveyed junior doctors. A total of 27 potential participants were contacted, of which 15 were female, nine male, three undisclosed.

Interested individuals contacted JS and gave informed consent. Participants were given the chance to ask JS questions about the research team and the study before interviews went ahead. Fifteen junior doctors (12 female, three male) were recruited.

Data collection

A semi-structured interview guide was developed by the research team and modified iteratively as data collection and analysis progressed. This guide aimed to capture participants' views, experiences, feelings and beliefs about working conditions and cultures which were perceived as stressful or distressing. The guide was informed by the existing literature [1, 3, 14], input from junior doctors on the study team as well as patient and public involvement (PPI) consultation exercises conducted before obtaining funding. Following conventions for semi-structured interviews [24], points from the topic guide were followed up with individualised questions exploring topics of interest and importance for each participant.

Interviews were conducted either on the telephone or via video call, from participants' homes or places of work. They took place between December 2020 and February 2021 – that is, during the second wave of the COVID-19 pandemic in the UK – and at a date and time that were convenient to the participants. A risk protocol was used to ensure that appropriate support from two senior GPs who were on the study team and/or Practitioner Health would be provided to participants in the event of the disclosure of suicidal ideation. The in-depth interviews were conducted by JS, a female PhD psychologist with extensive qualitative methods expertise. JS also recorded any pertinent observations in field notes following each interview. Interviews ranged from 29 minutes to 102 minutes in length (mean = 62.8 minutes).

For peer review only - http://bmjopen.bmj.com/site/about/guidelines.xhtml

For peer review only

The audio-recorded interviews were transcribed verbatim and checked for accuracy by JS before analysis. All transcripts were anonymised before discussion within the wider research team. Reflexive notes were recorded by researchers throughout the process.

Patient and public involvement and engagement

There are three junior doctors on the research team, all of whom consulted with other colleagues in the PPI team about the initial research idea and participated in analysis meetings. Five junior doctors gave feedback on the initial funding application, while four fed back on the protocol, topic guide and participant-facing documents.

Data analysis

Data were analysed by JS using reflexive thematic analysis [25, 26], in which themes highlight patterns of shared meaning united by a core concept. An inductive, explicit, critical realist approach was adopted since this was in line with the researchers' desire for a rich, data-driven analysis which demonstrated the interplay between events and participants' interpretations of those events [25]. Data saturation is not a relevant concept within this type of approach, in which it is accepted that each new participant adds fresh insights. Analysis began once all interviews had been conducted. Transcripts were analysed one by one using NVivo 12. As analysis progressed, a table of themes was generated and refined. Each new transcript led to new codes and themes being added or expanded. In addition, four members of the team, one of whom was a junior doctor and two of whom were academic GPs (RR, MB, AT, CCG), read and fed back on six of the 15 interviews. Their views and insights were collaboratively incorporated into the NVivo codes. JS then refined these codes to create relevant tables of themes once all interviews had been analysed and discussed. Analysis continued and deepened during the write-up, where shared meanings were generated and described for each theme [26].

Reflexivity

RR, the study PI, is epistemologically steeped in qualitative traditions underpinned by interpretivism and phenomenology, and is oriented by critical theory such as feminism. This

For peer review only - http://bmjopen.bmj.com/site/about/guidelines.xhtml

is likely to have influenced her interest in exploring why female doctors are more likely to experience distress.

JS, the lead analyst on this paper, is a qualitative health psychologist. She has an interest in in-depth, interpretative methods. She is white, cis-gendered, heterosexual and able-bodied. This heteronormative positioning is likely to have impacted her interviewing and analysis.

Both researchers have an interest in the systemic issues impacting individual NHS workers and are motivated by trying to find organisational – rather than individual – solutions for those workers.

The junior doctor (AT) and academic GPs (MB and CCG) who also contributed to analysis of the data have experienced and observed events during their professional lives which may have influenced how strongly they interpreted the data. Additionally, MB and CCG have a strong interest in mental health.

Findings

All fifteen participants discussed the impact of COVID-19 on their working conditions. Findings divided into three major themes: Challenges of working during the COVID-19 pandemic; Strategies for coping with the impact of COVID-19 on work; Positive impact of the COVID-19 pandemic on working practices.

See Table One for an overview of all relevant themes and subthemes.

For peer review only

For peer review only - http://bmjopen.bmj.com/site/about/guidelines.xhtml

Theme	Subtheme
Challenges of working during the Covid-19 pandemic *"patients were just dying in front of us so quickly and they were young" (P5)*	Personal impact: Helplessness in the face of trauma *"my sleep is awful again, I'm waking up, I think COVID hasn't helped" (P1)*
	Work-related impact: Change and uncertainty *"I gained 14 new patients who I'd not met before" (P6)*
Strategies for coping with the impact of Covid-19 on work *"So although I should have moved on from GP I ended up staying in GP so I was actually there for eight months." (P7)*	Limitations of personal strategies *"I cried a lot" (P14)*
	Organisational strategies *"we'd kind of share what we've learned" (P5)*
Positive impact of Covid-19 on working practices *"since COVID, things have improved slightly there's, um we have something called like the rest and recuperation hub." (P6)*	Positive new ways of working *"We almost looked forward to going to work" (P8)*
	Additional support and camaraderie *"they provided hot meals" (P5)*

Table one: Table of themes for junior doctors' experience of working during the Covid-19 pandemic

For peer review only - http://bmjopen.bmj.com/site/about/guidelines.xhtml

Challenges of working during the COVID-19 pandemic

Participants described challenges related to their work as junior doctors during the COVID-19 pandemic. Challenges were personal or work-related.

Personal impact: Helplessness in the face of trauma

Working as junior doctors during the COVID-19 pandemic affected participants' mental health. Throughout this theme, there is a sense that participants felt helpless and powerless as they strove to carry out their jobs in such unmapped territories.

One participant described the harmful impact of being exposed to death and suffering:

> *I'd seen [pause] a whole ward just emptied out and then refilled overnight, after people had just died. It was horrendous. Uh, I was like, "I need to talk to somebody about this or I'm just going to go home and cry". (P5, female)*

This participant's language – 'emptied out' and 'refilled' – suggests that the COVID-19 patients had become dehumanised for her; a mass of unwell bodies who were dying and being replaced in an almost mechanical manner. She was helpless to stop this flow of nameless bodies.

Participants felt helpless in the face of fears for their own safety and that of their loved ones. Initially, they were unsure of how to protect themselves or of the risk they might pose to their families:

> *…we had someone that we thought was, um, COVID, but it was very, very early on. And I remember being told off for wearing a mask. (P3, female)*

> *…we were worried about if we were taking home our clothes, if we were making other people sick, if we would get sick, it was an incredibly stressful working environment. (P5, female)*

For peer review only - http://bmjopen.bmj.com/site/about/guidelines.xhtml

As time went on, fears for personal safety came from different sources, with one participant reporting that her colleagues were not maintaining safety standards. However, as a junior doctor, she felt powerless to ask for this to change:

It's not patients, it's staff. I find that really stressful. Like you walk past an office and there might be two or three people sat in an office having a chat, all with their masks under their chin. […] I don't feel confident enough to knock on the window and be like guys, what are you doing? But I know that them doing it puts me more at risk and puts the patients at risk. […] You see stuff being wrong and you're like every day, like multiple times a day you're like do I say something, do I not say something? And you feel bad for not saying something. (P10, female)

This description of discomfort could be defined as moral injury; that is, the distress that occurs when a person witnesses or carries out an act that is contrary to their values. The participant felt uncomfortable and helpless however she responded.

Participants were powerless to switch off or rest when they got home from work:

You couldn't switch off, you never felt like you'd had, uh, done a good job. (P5, female)

…my sleep is awful again, I'm waking up, I think COVID hasn't helped with these sort of flashbacks. (P1, female)

Participants did not feel clinically supported in the new working environment caused by COVID-19, which led to further helplessness, fear and trauma. The lack of support could be practical:

I'm going to personally take responsibility for changing […] the big scary machine that I'm not trained in, and, uh, figure out how it works, whilst the patient is there trying to physically die in front of me, but so are five others, so oh well, no help is coming. (P5, female)

For peer review only - http://bmjopen.bmj.com/site/about/guidelines.xhtml
For peer review only

Some felt unsupported psychologically, with one participant appearing to feel that her needs were invisible to those who might support her:

> *…they got some psychologists who would be available and very occasionally they would come on the ward. [pause] And they would talk to the nurses. And that was it. No. It felt assumed to be on the nurses and people working in ITU and just ordinary junior doctors didn't [pause] didn't seem to matter. (P14, female)*

Another felt unsupported in terms of her physical health; her safety was compromised, meaning she was unable to protect herself:

> *…you will turn up on a ward and you will find out halfway through handover that they've had a positive case over the weekend. (P10, female)*

Additionally, a junior doctor whose family were overseas reported feeling unsupported by her hospital after contracting COVID-19:

> *…when I went to quarantine, I realised that no-one actually cares about you from the hospital? […] No-one called me! […] When I was very very sick, imagine that, if I had, if I had literally no-one. (P4, female)*

Work-related impact: Change and uncertainty

The work-related impact of working during a COVID-19 context centred around uncertainty and change. These included changes to workload, staffing levels, relationships with colleagues and patients, lack of support and uncertainty around new ways of working.

The junior doctors' workload grew significantly when COVID-19 hit, leading to further stressors. This led to a huge and stressful increase in one participant's responsibilities:

For peer review only - http://bmjopen.bmj.com/site/about/guidelines.xhtml

...on a Friday in the middle of the day when there was no consultant around [...] I gained 14 new patients who I'd not met before [...] that was a really stressful day. (P6, female)

Workload increased out of hours as well, as participants were constantly having to learn new facts about the virus and its management. The quote below demonstrates the doubt and pressure felt whilst trying to learn in the face of unmanageable amounts of new information:

So we were getting 20 emails a day, and every single one would have a red flag saying "vital, important, must read", and you'd worry you'd missed something [...] there's so much information, it was constant, and you couldn't switch off, because it would impact your job. (P5, female)

As workload rose, staffing levels, which had already been stretched, were adversely affected by further staff reductions due to illness or the need to self-isolate, demonstrating additional change and uncertainty:

So it's very very short-staffed because a lot of the people are self-isolating, ill with COVID, or just because you know they've worked already five or six days in a row, and obviously they're quite tired and they have to take a break. (P12, male)

The additional workload changed working relationships in various ways. Participants reported that colleagues became irritable or verbally aggressive due to increased stress:

I think everyone got a little bit more [pause], um, maybe snippy? With each other? 'Cause we were all are very stressed and anxious. (P3, female)

For peer review only - http://bmjopen.bmj.com/site/about/guidelines.xhtml
For peer review only

…a registrar wearing an MF53 mask[1] and the consultant laying into him basically shouting at him that […] he was depriving someone else who actually needed this mask […] emotions were running high because people were scared. (P14, female)

One trainee, based in general practice, reported that patients had become abusive during telephone appointments, potentially dehumanising their doctors:

…sometimes people lose sense of the fact that it's another human being on the end of the phone with them. And you're already dehumanised a little bit as a doctor because people expect you to be more than, more than human. And when you then couple that with someone just being this kind of like faceless voice on the end of the phone, especially when people are scared or something like that, it just there's that heightened level of aggression. (P7, female)

That participant also reported finding the change to telephone appointments clinically challenging and risky in terms of being able to diagnose patients accurately.

I don't think you realise how much you rely on seeing someone in front of you to know how well they are. And talking to someone over the phone it just feels a lot more dangerous. (P7, female)

Compounding these changes which made participants' working lives harder was the fact that it also became harder to speak with and get support from peers due to the safety measures:

Um, and now with COVID where you're only allowed, like, two people in each room, it, it's very difficult to, um, socialise and talk. (P8, female)

As junior doctors in training, participants also found the uncertainty around changes to rotas and exams challenging:

[1] This is a full face, military style of mask

For peer review only - http://bmjopen.bmj.com/site/about/guidelines.xhtml

> *…a fair amount of uncertainty and the problem this time is that, ah, a lot of courses are still going ahead, exams are still going ahead, but we've been moved onto emergency rotas with a week's notice. (P8, female)*

The junior doctors were often expected to work in different specialities or locations from those which they had been allocated to pre-pandemic. Constant anxiety due to uncertainty about redeployment was reported:

> *…anxious and uncertain about whether that was going to happen and would sort of check my emails pretty consistently to see whether that was actually whether that was going to be um delayed or stopped because of COVID redeployment. (P6, female)*

The pandemic meant that new ways of working were quickly developed and implemented. Trying to adjust to these changes was another challenge. One trainee in psychiatry talked about the potential stress and impact on patient care of working from home:

> *…you've not got those people around you to bounce things off, so you might get an email and it might be quite an anxiety-inducing email because it might say someone's suicidal, you need to see them, and you're thinking, oh, I can't see them, erm, and normally, kind of in an office you'd just be able to ask, can anyone else see them? (P2, female)*

Strategies for coping with the impact of COVID-19 on work

Participants described both personal and organisational strategies for coping with the above challenges.

For peer review only - http://bmjopen.bmj.com/site/about/guidelines.xhtml

Inadequate personal coping strategies

Emotion-focused and problem-focused coping strategies were utilised for dealing with the challenges of COVID-19. However, there was a sense that these personal coping strategies, which might have been adequate before COVID-19, were not enough to protect participants from the impact of working during the pandemic.

Emotion-based coping strategies included crying:

> *So I cried a lot outside. Because it was getting warmer so you could go outside. Hug a tree, cry. (P14, female)*

Stoicism was used by some, although this latter strategy suggested a sense of resignation, illustrated by P8's rhetorical and hopeless question:

> *And [pause] and in a way it didn't really matter that our rota changed, because there was nothing else to do? (P8, female)*

A sense of powerlessness combined with acceptance was perceived to have impacted the profession as a whole:

> *I think erm you know everyone's a bit more sort of resigned to things now and it feels like we've sort of erm entered a collective sort of depressive state of acceptance. (P9, male)*

We can see that these personal, emotion-based coping strategies had their limits when employed during the COVID-19 pandemic.

For peer review only - http://bmjopen.bmj.com/site/about/guidelines.xhtml

Problem-focused strategies were perhaps more effective. One participant volunteered to take on the work of calling relatives to let them know their loved ones were very sick, perhaps to be able to provide a more personal input to such a traumatic situation.

> *I used to volunteer to kind of be the person making those phone calls, cos it was, it felt like you were able to do something about it at least? It wasn't that sort of like, "I put lines in people and hopefully", and then just watching them die. (P5, female)*

Another participant agentically took control of her situation by arranging more support for herself, perhaps in response to the helplessness described in the previous theme:

> *…when lockdown came back in […] I noticed that like I was feeling low so I referred myself to the Let's Talk Wellbeing, erm which is like the community, CBT, GP, self-referral system. And I found that really helpful erm so that kind of stopped me spiralling. (P10, female)*

Organisational strategies

Just as participants found ways to cope with the challenges of COVID-19, so did the organisations and teams for whom they worked, with some trusts and teams demonstrating collaborative, flexible thinking One participant reported flexibility in terms of working from home for colleagues who had to self-isolate:

> *…most of the places have let the person sort of choose whether they you know, if it's your child that's got a fever and actually you know you're isolating and could do things then that's fine. But if you're poorly then you're poorly and that's fine. (P11, female)*

Another described the need for her team to make pragmatic decisions about how to treat COVID-19 patients:

For peer review only - http://bmjopen.bmj.com/site/about/guidelines.xhtml

So if someone was clearly dying, they would [pause] be stepped down to a normal ward because on a normal ward they could at least have a visitor for one hour a day. (P14, female)

A third participant reported that her team pulled together to help one another in the new circumstances:

…there'd be so many [emails] even coming in during our shift, we'd divide it up, so we'd say, "you read these five, I'll read these five, you read these five, and then I'll read these five", and then we'd kind of share what we've learned from them. (P5, female)

Positive impact of COVID-19 on working practices

Participants reported that working as junior doctors during the COVID-19 pandemic had some positive impacts. These included new and less bureaucratic ways of working as well as additional support and camaraderie.

Positive new ways of working

Positive changes revolved around a less bureaucratic way of working, which included consistent teams, longer rotations and less red tape.

Several participants reported that they were now working in a consistent team, rather than regularly working with new colleagues. This was experienced as positive:

So normally, you're kind of working with somebody new every day almost. But we worked in teams that didn't rotate, so you had […] this team that you worked with very intensely for those four months as well, and that support structure was really good. (P5, female)

…we got really to know each other, we had a little social WhatsApp group where we'd, like, post pictures of the cakes we were gonna bring in, you know, everyone bought in food. We almost looked forward to going to work because you were like, oh, my buddies are there. (P8, female)

A sense of being part of a team and able to enjoy work comes across in the above quote, where cake and conversation bring some positivity to the bleak picture painted thus far.

Rotations were paused for many junior doctors. Although this could lead to uncertainty, as reported in an earlier theme, it also had some potentially positive impacts:

So we were on the first rotation for four months and then the second for eight months […] Um, so, I guess it would have depended on what ward you got stuck on? [interviewer laughs] Um, I got stuck on one of the nice placements, I really enjoyed myself on the ward. (P3, female)

Various practical changes to working patterns were also experienced as positive. These included simple factors such as the ability to work from home and reduced red tape:

…just get away and do something relaxing, even if it's just go for a walk around the local canal and come back on a lunchtime is so much more achievable when you're working from home. So I think that's been really good. (P2, female)

…they say oh, we want you to travel to a hospital on your day off to show us your passport and your GMC certificate. And it's like I've been – doing this for 10 years. I've worked for you six times! Like, you've got my details [both laugh]. And that's one thing where COVID has been really good because now they do it online and I'm like, why couldn't you have always done this? (P8, female)

One participant even appeared to cite COVID-19 as a motivator for returning to work at the NHS after time in another career:

For peer review only - http://bmjopen.bmj.com/site/about/guidelines.xhtml

…then COVID came and I wanted to come back to medicine anyway so I thought okay fine then let's just crack on with erm with the NHS. (P15, female)

Additional support and camaraderie

Some participants reported that new supportive measures, such as additional facilities, had been put in place by their workplaces:

…they provided hot meals, which, at the beginning, when there were huge queues at the supermarket, and we were working 12-hour shifts, five days a week, and, um, [pause] and it was unpredictable whether you could kind of get food, because there were a lot of shortages and things. (P5, female)

And since COVID, things have improved slightly, there's, um we have something called like the rest and recuperation hub, which is like a room erm that does free teas and coffees and a few snacks […] you go there on your breaks to relax. (P6, female)

Another participant reported that her hospital made an effort to offer junior doctors support, although this was against the backdrop of a toxic working environment:

I'd say the culture's getting worse except for the fact that they send an e-mail out every now and again with some contact numbers [for support services] and that's what COVID has done. (P1, female)

It should be noted that the reports of improved support were tempered – note that participants reported 'slight' improvements to a culture that was also 'getting worse'.

Discussion

Fifteen distressed junior doctors were interviewed between December 2020 and February 2021 about their perceptions of stress and distress in their workplace cultures. All

For peer review only - http://bmjopen.bmj.com/site/about/guidelines.xhtml

participants discussed how COVID-19 impacted their experiences. Looking at our themes as a gestalt, we suggest that the helplessness that arose due to the trauma of working during the pandemic meant that individual coping strategies which may have been more beneficial during less unusual times fell short, something that often went unrecognised by employers. To compound this, participants were also not sufficiently supported either practically or psychologically during this time. This may have led to feeling powerless and resigned in the face of difficult circumstances for which they were unprepared. Additionally, we recommend that the positive lessons highlighted in this paper are followed in the long term.

Helplessness was commonly reported whilst working during COVID-19. Specifically, one participant described how traumatic it was to see so many patients dying. Others [8, 10, 27] have cited grief and managing such large numbers of patient deaths as especially challenging. We suggest that newer junior doctors might need extra support to process grief in such exceptional circumstances, for which they had not been trained. This might especially be the case for younger doctors [28] and female doctors [29, 1] since it has been shown that these groups, who made up the majority of participants in the current study, are potentially more vulnerable to depression, stress and suicidal thoughts.

Another participant reported an experience of moral injury following the unsafe behaviour of other staff members. Moral injury due to redeployment away from long-term patients [15] and concerns about letting patients down [8] during COVID-19 has also been reported. Our findings add an additional perspective, demonstrating that moral injury can also arise due to staff members neglecting safety protocols.

Adding to these traumatic personal experiences, participants reported that while their workload rose, staffing levels often decreased. Previous research has shown that, following austerity [30], UK HCPs were already working in an under-resourced environment and that additional workload is a potent stressor [21, 31-33]. Crises such as COVID-19 further emphasise the need for extra resources for our healthcare system, echoing the recommendations of the 2009 Boorman report [34], which have been widely neglected [6]. It is often harder for frontline workers to take breaks during a pandemic [5, 10], adding to the potential for burnout since longer working hours are a risk factor [29]. Cubitt and

For peer review only - http://bmjopen.bmj.com/site/about/guidelines.xhtml

colleagues [5] have highlighted the need for rotas that enable well-being rather than merely being resilient; that is, containing the bare minimum number of doctors per shift. Qualitative research such as the current study adds depth to these recommendations by demonstrating the instability, lack of support and powerlessness that distressed HCPs faced during this time.

Participants felt unsupported whilst working in these new, traumatic circumstances, a finding reflected elsewhere [7, 10, 11]. For example, one participant who needed psychological support intimated that she felt invisible. Whilst the needs of others – nurses and non-medical staff – were considered, her needs were assumed not to exist, demonstrating the powerlessness of the junior doctors in this situation. If you cannot be seen, you cannot be helped.

The need for extra training and support for junior doctors during pandemics has been reported [15]. We echo this recommendation and would add that support could come from good leadership which recognises the challenges staff face, a feeling of being valued within a team and by addressing the practical and physical limitations junior doctors frequently experience, such as poor 'on call' accommodation and access to regular meals. We suggest that employers often fail to recognise the limitations of individual coping strategies, both during crises such as the pandemic and in less unusual times.

Participants used various strategies to attempt to cope with working during COVID-19. Emotion-focused strategies such as crying were reported in our study although these strategies often appeared limited in usefulness. At times, the stoicism reported by participants in the current study verged on learned helplessness, demonstrating that personal coping strategies alone are not enough, and that coping is not guaranteed in a healthcare crisis when doctors are already stressed and distressed. Various individualised coping strategies have been suggested, including healthy eating, attending training, going to therapy, support networks [6] and making use of 'wobble rooms' [15]. However, San Juan and colleagues [6] recognised that finding time for these activities might be difficult, particularly during a time of crisis.

For peer review only - http://bmjopen.bmj.com/site/about/guidelines.xhtml

Owens et al. [30] state that if we are continually asking our HCPs to behave heroically in exceptional circumstances, we are inviting burnout. Indeed, it could be posited that encouraging such strategies places the responsibility for managing the unmanageable with individuals, rather than the system [6]. It is argued that, in our neo-liberal culture, responsibility for wellbeing is often placed on the individual, exonerating the state and systems for the wellbeing of workers [30, 35, 36]. This can be seen in the use of the term 'resilience', which places responsibility for managing the unmanageable on the shoulders of individuals, rather than organisations [35, 36]. Therefore, in line with San Juan et al. [6], we recommend a focus on organisational, rather than the personal, coping strategies. Those organisational strategies could, as seen in our findings include flexibility and better organisational, managerial and peer-support through teamwork and collaboration as well as addressing the practical workplace issues which could lead to HCPs feeling physically safe and cared for. Vulnerable junior doctors need organisational support especially, although not exclusively, during crises like COVID-19. However, the emphasis continues to be on the individual [37, 38].

Participants reported several potentially positive impacts of working during the pandemic, a novel finding. These included working in more consistent teams. San Juan and colleagues [6] have similarly reported that consistent teams are helpful for HCP, while inconsistent teams make it harder for junior doctors to seek support [14], increasing stress and vulnerability to mental ill-health [21]. As such, we recommend that, where possible, policymakers consider the use of consistent teams for junior doctors going forward. The beneficial impact of a reduction in bureaucracy reported by one participant appears to be another novel finding. We would suggest any such reductions should be maintained after the pandemic ends, with a potential reduction in time pressures for junior – and senior – doctors as well as other healthcare workers.

Participants stated that some new supportive measures, such as rest hubs, had been put into place during COVID-19. Such spaces have been deemed helpful by other researchers [7, 10, 15], although there are anecdotal reports that many of these spaces have now been closed as hospitalisations from COVID-19 reduce. In contrast, HCPs in other studies have

For peer review only - http://bmjopen.bmj.com/site/about/guidelines.xhtml

reported that the extra strain on the system meant that there were fewer places than usual to shower, rest or relax with colleagues [5, 6].

In line with our findings, Vindrola-Padros et al. [7] reported that there was extra signposting towards support during COVID-19; however, there was not often time to engage with this support. Additionally, it has been anecdotally reported that much of this support has been withdrawn now. This adds further weight to the notion that systemic, holistic changes are needed to support NHS staff, rather than focusing the responsibility for change on individuals [6]. We suggest that such limited responses from employers may have contributed to the feelings of resignation described by some of our participants.

Limitations

This study has various strengths, including being the first qualitative paper (to our knowledge) to explore the experiences of junior doctors during COVID-19. Our data was collected during the pandemic and we utilised in-depth, collaborative thematic analysis. However, despite these strengths, the paper has several limitations. We did not recruit these participants specifically to talk about the Covid-19 pandemic. Rather, the timing of the study meant that the topic arose naturally. As such, the interview guide could have been designed to ask participants more thoroughly about these experiences. Additionally, some of the junior doctors had more experience of working with COVID-19 patients than others, meaning some participants are better represented in this paper than others. Further, there is a notable gender disparity, with a higher proportion of female doctors taking part. More female (n=12) participants volunteered than males (n=3). The increased willingness of female participants to speak about their experiences may be associated with evidence indicating that female doctors are more likely to experience distress. Sadly, this group are also more likely to kill themselves [1]. The higher proportion of female participants may also reflect gendered help-seeking behaviour for mental ill-health, evidenced in the wider population [38], as well as the fact that female doctors are more likely to take part in research than their male counterparts [39]. Finally, it should be reiterated that this was a purposive sample of particularly distressed junior doctors, albeit taken from a wider sample

For peer review only - http://bmjopen.bmj.com/site/about/guidelines.xhtml

in which distress was widely reported, and so our findings are not intended to be generalised to all junior doctors.

Conclusions and recommendations

We conclude that junior doctors working during the COVID-19 pandemic faced multiple stressors and used various coping mechanisms to deal with these, with greater or lesser degrees of success. Several unexpected benefits of this period arose, including new ways of working and additional support and camaraderie. We believe that the responsibility for alleviating the stress and distress of junior doctors working during times of stress lies with organisational employment issues and systemic workforce gaps, rather than with individuals. As such, we recommend system-wide changes, such as improved communication strategies, increased flexibility around home-based working, addressing the physical limitations of the working conditions many junior doctors experience and more supportive and compassionate leadership. Additionally, we suggest that, where possible, junior doctors are assigned to consistent teams, with the opportunity for appropriate psychological support where indicated.

Author contributions: RR, JS, CCG, MB, AD, KT, AG, AT, MvH, LA, JM – substantial contributions to conception and design; approval of final version to the published.

RR, JS, CCG, MB, AT, KT, AD, AT – acquisition, analysis or interpretation of data; drafting the article or revising it critically for important intellectual content.

Acknowledgements: The authors would like to thank all junior doctor participants who kindly gave their time to share their experiences of working in the NHS. We also wish to thank members of the PPIE group who provided valuable input throughout the study.

Data sharing: This study has not received ethical approval to share confidential data with any third party other than the research team.

For peer review only

Ethical approval: Ethical approval was granted by the University of Birmingham and Health Research Authority (reference number: 19/HRA/6579)

For peer review only

For peer review only - http://bmjopen.bmj.com/site/about/guidelines.xhtml

References

1. Dutheil, F., et al. *Suicide among physicians and health-care workers: A systematic review and meta-analysis*. PloS one. 2019. **14**(12): p. e0226361

2. Petrie, K., et al. *Interventions to reduce symptoms of common mental disorders and suicidal ideation in physicians: A systematic review and meta-analysis*. The Lancet Psychiatry. 2019. **6**(3): 225-34.

3. Brooks, S. K., et al. *Review of literature on the mental health of doctors: Are specialist services needed?* Journal of Mental Health. 2011. **20**(2): 146-56.

4. Chopra, S. S. et al. *Physician Burnout*. JAMA. 2004. **291**(5): 633–33.

5. Cubitt, L. J. et al. *Beyond PPE: A mixed qualitative-quantitative study capturing the wider issues affecting doctors' well-being during the COVID-19 pandemic*. BMJ Open. 2021. **11**(3): e050223.

6. San Juan, N. V., et al. *Healthcare Workers' mental health and wellbeing during the COVID-19 pandemic in the UK: Contrasting guidelines with experiences in practice*. MedRxiv. 2020; advanced online.

7. Vindrola-Padros C., et al. *Perceptions and experiences of healthcare workers during the COVID-19 pandemic in the UK*. BMJ Open. 2020; **10**(11): Advanced online: e040503.

8. Liberati E., et al. *A qualitative study of experiences of NHS mental healthcare workers during the Covid-19 pandemic*. BMC Psychiatry. 2021. **21**(1): 1-12.

9. Sun, N., et al. *A qualitative study on the psychological experience of caregivers of COVID-19 patients.* American Journal of Infection Control. 2020. **48**(6): 592-8.

10. Blake, H., et al. *COVID-well study: Qualitative evaluation of supported wellbeing centres and psychological first aid for healthcare workers during the COVID-19 pandemic.* International Journal of Environmental Research and Public Health. 2021. **18**(7): 3626-59.

11. Newman K. L., et al. *Experiences and emotional strain of NHS frontline workers during the peak of the COVID-19 pandemic*. International Journal of Social Psychiatry. 2021; advanced online 00207640211006153.

12. Cawcutt K. A., et al. *Fighting fear in healthcare workers during the COVID-19 pandemic*. Infection Control & Hospital Epidemiology. 2020. **41**(10): 1192-3.

13. BMA, *https://www.bma.org.uk/advice-and-support/international-doctors/life-and-work-in-the-uk/toolkit-for-doctors-new-to-the-uk/doctors-titles-explained.* 2020.

For peer review only

For peer review only - http://bmjopen.bmj.com/site/about/guidelines.xhtml

14. Fox F. E., et al. *Junior doctors' experiences of personal illness: A qualitative study*. Medical Education. 2011. **45**(12): 1251-61.

15. Coughlan C., et al. *COVID-19: Lessons for junior doctors redeployed to critical care*. Postgraduate Medical Journal. 2021. **97**(1145): 188-91.

16. Lion P., et al. *ENT trainees' experience of redeployment during the coronavirus disease 2019 pandemic: A qualitative study*. The Journal of Laryngology & Otology, 2021. 1-13.

17. Aziz, A., et al. *Rapid transformation of the medical on-call rotas to deal with the COVID-19 pandemic: A case study*. Future Healthcare Journal. 2021; **8**(1): e36.

18. Seifman M. A., et al. *COVID-19 impact on junior doctor education and training: A scoping review*. Postgraduate medical journal. 2021.

19. Rimmer, M. P., et al. *Provision of obstetrics and gynaecology services during the COVID-19 pandemic: A survey of junior doctors in the UK National Health Service*. BJOG: An International Journal of Obstetrics & Gynaecology. 2020. **127**(9): 1123-8.

20. Riley, R., et al. *Protective factors and sources of support in the workplace as experienced by UK foundation and junior doctors: A qualitative study*. BMJ Open. 2021. **11**(6): e045588.

21. Riley, R., et al. *Sources of work-related psychological distress experienced by UK-wide foundation and junior doctors: A qualitative study*. BMJ Open. 2021. **11**(6): e043521.

22. Osman, A., et al. *The depression anxiety stress scales—21 [DASS-21]: Further examination of dimensions, scale reliability, and correlates*. Journal of clinical psychology. 2012. **68**(12): 1322–1338.

23. Paykel, E. S., et al. *Suicidal feelings in the general population: A prevalence study.* The British Journal of Psychiatry. 1974. **214**(582): 460–9.

24. King N. and C. Horrocks, *Interviews in Qualitative Research*. 2010, London: Sage.

25. Braun V. and V. Clarke, *Using thematic analysis in psychology*. Qualitative research in psychology, 2006. **3**(2): 77-101.

26. Braun V. and V. Clarke, *Reflecting on reflexive thematic analysis*. Qualitative Research in Sport, Exercise and Health, 2019. **11**(4): 589-97.

27. Johnson, S., et al. *Impact on mental health care and on mental health service users of the COVID-19 pandemic: A mixed methods survey of UK mental health care staff*. Social Psychiatry and Psychiatric Epidemiology, 2021. **56**(1): 25-37.

For peer review only - http://bmjopen.bmj.com/site/about/guidelines.xhtml

28. Kinman G. and K. Teoh K, *What could make a difference to the mental health of UK doctors? A review of the research evidence*. 2018.

29. Kinman, G., et al. *The mental health and wellbeing of nurses and midwives in the United Kingdom*. Royal College of Nursing Foundation and the Society for Occupational Medicine, 2020.

30. Oliver, D., *The NHS's understaffing is its Achilles' heel*. BMJ: British Medical Journal (Online), 2017. 357.

31. Boorman, S. *NHS Health and well-being review: Interim report*. NHS Health and Wellbeing Review, 2009.

32. Amoafo, E., et al. *What are the significant factors associated with burnout in doctors?* Occupational Medicine, 2015; **65**(2): 117-21.

33. Pollock, A., et al. *Interventions to support the resilience and mental health of frontline health and social care professionals during and after a disease outbreak, epidemic or pandemic: A mixed methods systematic review*. Cochrane Database of Systematic Reviews, 2020. 11.

34. Sood, A., et al. *Stress management and resilience training among department of medicine faculty: A pilot randomized clinical trial*. Journal of general internal medicine, 2011. **26**(8): 858-61.

35. Royal College of Surgeons of Edinburgh, https://committees.parliament.uk/writtenevidence/11033/html/, 2020.

36. Joseph, J., *Resilience as embedded neoliberalism: A governmentality approach*. Resilience, 2013. **1**(1): 38-52.

37. Traynor, M. *Guest editorial: What's wrong with resilience*. Journal of Research in Nursing, 2018. **23**(1): 5-8.

38. Seidler, Z. E., et al. *The role of masculinity in men's help-seeking for depression: A systematic review*. Clinical Psychology Review, 2016. **49**: 106-18.

39. Cull, W. L., et al. *Response rates and response bias for 50 surveys of pediatricians.* Health Services Research, 2005. **40**(1): 213-26.

For peer review only - http://bmjopen.bmj.com/site/about/guidelines.xhtml

Consolidated criteria for reporting qualitative studies [COREQ]:

Developed from: Tong, A., Sainsbury, P., & Craig, J. [2007]. Consolidated criteria for reporting qualitative research [COREQ]: a 32-item checklist for interviews and focus groups. *International journal for quality in health care*, *19*[6], 349-357.

**No. Item**	**Guide questions/description**	**Reported on page #**
**Domain 1: Research team and reflexivity**		
Personal characteristics		
1. Interviewer/ facilitator	Which author/s conducted the interview or focus group?	Page 11
2. Credentials	What were the researcher's credentials? E.g. PhD, MD.	Page 11
3. Occupation	What was their occupation at the time of the study?	Page 11
4. Gender	Was the researcher male or female?	Page 11
5. Experience and training	What experience or training did the researcher have?	Page 11
Relationship with participants		
6. Relationship established	Was a relationship established prior to study commencement?	Page 11
7. Participant knowledge of interviewer	What did the participant know about the researcher? E.g. personal goals, reasons for doing the research.	Page 11
8. Interviewer characteristics	What characteristics were reported about the interviewer/facilitator? E.g. bias, assumptions, reasons and interests in the research topic	Page 12
**Domain 2: study design**		
Theoretical framework		
9. Methodological orientation and theory	What methodological orientation was stated to underpin the study? E.g. grounded theory, discourse analysis, ethnography, phenomenology, content analysis	Page 12

For peer review only

Participant sampling		
10. Sampling	How were participants selected? E.g. purposive, convenience, consecutive, snowball	Page 10
11. Method of approach	How were participants approached? E.g. face-to-face, telephone, mail, email	Page 10
12. Sample size	How many participants were in the study?	Page 11
13. Non-participation	How many people refused to participate or dropped out? Reasons?	None
Setting		
14. Setting of data collection	Where was the data collected? E.g. home, clinic, workplace	Page 11
15. Presence of non-participants	Was anyone else present besides the participants and the researchers?	No
16. Description of the sample	What were the important characteristics of the sample? E.g. demographic data, date	Page 11
Data collection		
17. Interview guide	Were questions, prompts, guides provided by the authors? Was it pilot tested?	Page 11
18. Repeat interviews	Were repeat interviews carried out? If yes, how many?	No
19. Audio/visual recording	Did the research use audio or visual recording to collect the data?	Page 11
20. Field notes	Were field notes made during and/or after the interview or focus group?	Page 11
21. Duration	What was the duration of the interviews or focus group?	Page 11
22. Data saturation	Was data saturation discussed?	Page 12
23. Transcripts returned	Were transcripts return to participants for comment and/or correction?	No, due to lack of resources
**Domain 3: analysis and findings**		
Data analysis		
24. Number of data coders	How many data coders coded the data?	Page 12
25. Description of the coding tree	Did authors provide a description of the coding tree?	No
26. Derivation of themes	Derived from the data?	Page 12

For peer review only

27. Software	What software, if applicable, was used to manage the data?	Page 12
28. Participant checking	Did participants provide feedback on the findings?	No, due to lack of resources
Reporting		
29. Quotations presented	Were participant quotations presented to illustrate the themes/findings? Was each quotation identified? E.g. participant number	Pages 13-22
30. Data and findings consistent	Was there consistency between the data presented and the findings	Yes, see sages 13-22
31. Clarity of major themes	Were major themes presented in the findings?	Yes
32. Clarity of minor themes	Is there a description of diverse cases or discussion of minor themes?	Yes

For peer review only
